# Flowering time in banana (*Musa spp*.), a day neutral plant, is controlled by at least three *FLOWERING LOCUS T* homologues

**DOI:** 10.1038/s41598-017-06118-x

**Published:** 2017-07-19

**Authors:** Akhilesh K. Chaurasia, Hemant B. Patil, Bal Krishna, V. R. Subramaniam, Prafullachandra V. Sane, Aniruddha P. Sane

**Affiliations:** 1 Plant Molecular Biology Lab, Jain R&D Lab, Jain Hills, Jain Irrigation Systems Limited, Jalgaon, 425001 India; 2 Plant Gene Expression Lab, CSIR-National Botanical Research Institute, Rana Pratap Marg, Lucknow, 226001 India

## Abstract

Banana is an important day neutral food crop with a long flowering/fruiting cycle that is affected by hot summers or cold winters in different places. Manipulating its life cycle requires an understanding of its flowering time machinery to bypass these stresses. Twelve *FLOWERING LOCUS T* (*FT*) and two *TWIN SISTER OF FT* (*TSF*) members were isolated from banana and their organization and expression pattern studied during development in two varieties that differ in flowering time namely Grand Nain (AAA genotype) and Hill banana (AAB genotype). The expression of at least 3 genes namely *MaFT1*, *MaFT2* and *MaFT5* (and to some extent *MaFT7*) increases just prior to initiation of flowering. These four genes and five others (*MaFT3*, *MaFT4*, *MaFT8*, *MaFT12* and *MaTSF1* could suppress the delayed flowering defect in the Arabidopsis *ft*-*10* mutant and induce early flowering upon over-expression in the Col-0 ecotype. Most genes are diurnally regulated and differentially expressed during development and in various vegetative and reproductive tissues suggesting roles besides flowering. Subtle amino acid changes in these FT/TSF-like proteins provide interesting insights into the structure/function relationships of banana FTs vis-à-vis Arabidopsis. The studies provide a means for manipulation of flowering in banana for better management of resources and to reduce losses through abiotic stresses.

## Introduction

One of the most crucial phases in development in flowering plants is the decision to flower. The timing of flowering has a major influence on plant fitness. Flowering is controlled by several external factors such as photoperiod, temperature, abiotic stresses and internal factors like hormone levels, C/N ratios and age of the plant^1–4^. A complex set of interactions between the internal and external cues determines when the plant transitions from the vegetative to the reproductive phase.

Members of the Phosphatidyl Ethanolamine Binding Protein (PEBP) family include two groups of proteins with opposing functions namely, *FLOWERING LOCUS T* (*FT*) and *TERMINAL FLOWER1* (*TFL1*) that play key roles in the regulation of flowering time in plants^5–9^. A paralogue of *FT*, *TWIN SISTER OF FT* (*TSF*) also regulates flowering in *Arabidopsis* although it works redundantly with *FT* ^10^. Once conditions conducive to flowering are perceived by the plant, these signals activate FT expression in leaves. The FT product, now believed to be the elusive ‘*florigen*’, is translocated to the apex where it interacts with FD and, in monocots, also with 14-3-3 to form the ‘Florigen Activation Complex’ (FAC)^11–13^. This complex activates the expression of downstream targets such as the floral meristem identity genes, *LEAFY* and *APETALA1*, leading to flowering^2, 14–16^. *FT* not only acts in photoperiodic control of flowering but also controls flowering in day neutral plants like tomato^17^ as well as in plants that require vernalization^18, 19^.

The FT/TSF members of the PEBP family have been identified in all flowering plants that have been studied so far. In dicots such as Arabidopsis^10^, tomato^20^, Poplar^21^, sugarbeet^22^, apple^23^, etc. this family consists of one to six members. In monocots such as maize^24^, rice^25^, barley^26^, onion^27^, wheat^28^ etc. the FT/TSF sub-family is much larger with 6–15 members. Although initially implicated in floral transitions, FT members are now known to be involved in a range of developmental processes such as lateral shoot outgrowth and plant architecture^17, 29–31^, stomatal control^32^, bud set and photoperiodic growth control^33–35^, inflorescence meristem stabilization^36^, bulb formation^27^ and tuberization^37^. Thus, the FT family appears to have a far more versatile role in development than previously thought (reviewed by Pin and Nilsson, 2012)^38^.

Banana (both plantains and dessert banana) is one of the most important food and fruit crops in the world particularly in the developing world because of its year round availability as a relatively cheap source of carbohydrates and minerals. Dessert banana is a triploid, sterile plant, propagated vegetatively through tissue culture or ratoons. It is a tropical, day neutral plant, typically flowering in 7 to 9 months followed by an additional 2–3 months for fruit harvest. The long life cycle predisposes it to abiotic stresses such as high tropical summer temperatures coupled with reduced water availability or low winter temperatures with occasional frost that affect yields. Manipulation of the flowering time in banana is thus of primary interest so as to shorten the vegetative phase and thereby conserve water particularly during the hot tropical summers or reduce frost injury in winters.

Currently, little is known about the transition to flowering in this day neutral, economically important fruit plant. In an effort to address this, 14 FT/TSF-like genes in banana were isolated and nine were shown to functionally complement the Arabidopsis *ft*-*10* mutant. At least three of these namely, *MaFT1*, *MaFT*2 and *MaFT5* appear to be correlated with induction of flowering in banana.

## Results

### Banana genome contains 14 FT/TSF-like genes

**Figure 1:**
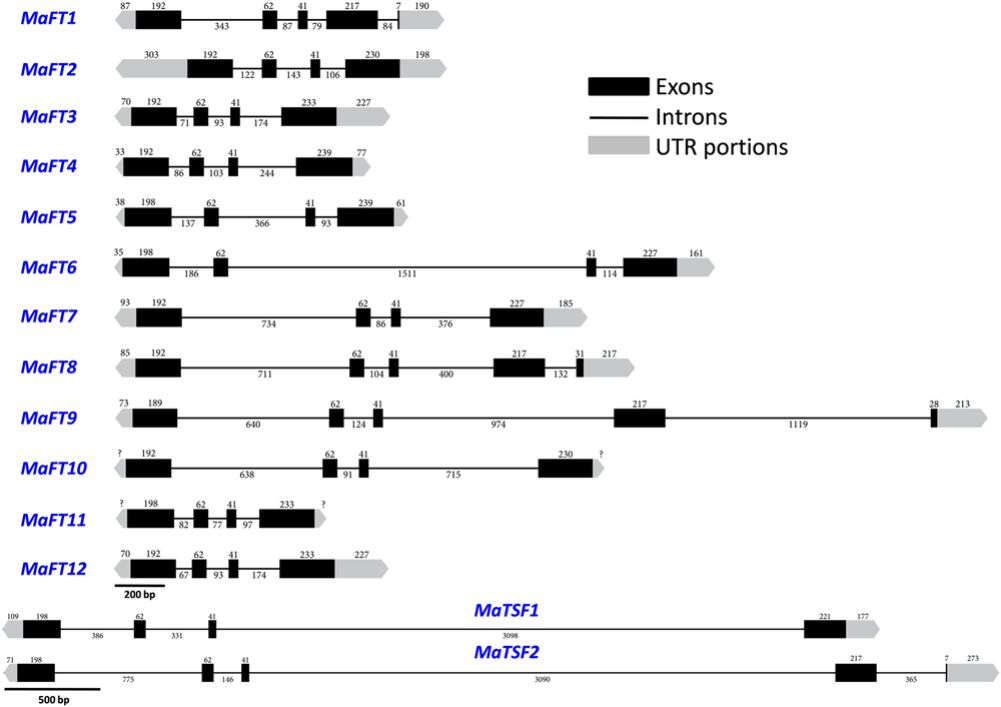
Schematic representation of the genomic organization of *FT*/*TSF*-like genes of banana. The black boxes represent different exons while the lines represent introns. The 5′ and 3′ UTRs are shown as grey pentagons. Numbers represent the lengths (bp) of exons, introns (shown below the lines) and UTRs (shown above the boxes).

A total of 14 FT/TSF-like genes were identified from banana (Grand Nain) using a combination of 5′/3′RACE on pooled cDNA of different tissues as well as by genome walking on banana genomic DNA library. The complete genomic sequences of all 14 genes (named *MaFT1*-*MaFT12*, *MaTSF1* and *MaTSF2*) with intron and exon boundaries are shown in Fig. 1. Full length cDNAs could be obtained for 13 genes, *MaFT10* being the exception.

The coding sequences of all 14 genes were largely conserved, ranging from 516 to 540 bp with the second and third exons being invariant in size. The genomic structure of FT/TSF-like genes, however, varied a lot due to intronic size variations with the largest intron of 3098 bp being present in *MaTSF1*. Interestingly, while the FT/TSF-like genes in most plants typically contain a conserved three intron structure, *MaFT1*, *MaFT8*, *MaFT9* and *MaTSF2* contained an additional fourth intron that split the fourth exon into two, thus changing the termination codon from that predicted in the database. To date, the presence of a fourth intron in *FT* like genes has only been reported in *Gypsophila paniculata*, a dicot^39^, but not in monocots.

### The MaFT/TSF genes are distributed across the different chromosomes

**Figure 2:**
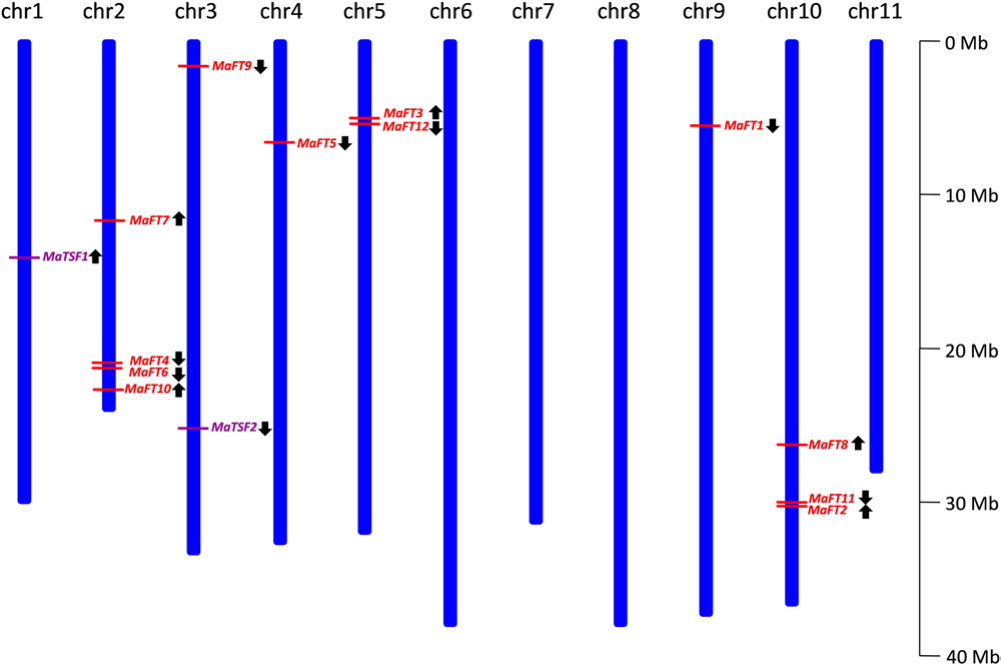
Chromosome distribution of banana *FT* and *TSF*-like genes. The red lines indicate the position of the gene and black arrows indicate the direction of transcription.

Using the available published banana sequence of DH-Pahang variety^40^, the 14 FT/TSF-like genes were localized on different chromosomes (Fig. 2, Supplementary Table S1). Four of these namely *MaFT4*, *MaFT6*, *MaFT7* and *MaFT10* were localized on chromosome 2 while *MaFT2*, *MaFT8* and *MaFT11* were localized on chromosome 10. Of these, *MaFT2* and *MaFT11* (facing each other) were separated by a distance of only 2515 nt. The two most similar FTs, *MaFT3* and *MaFT12*, were also located close to each other on chromosome 5, separated by 148328 nt. *MaFT9* and *MaTSF2* were located on chromosome 3 while *MaTSF1*, *MaFT5* and *MaFT1* were located on chromosomes 1, 4 and 9 respectively.

**Figure 3:**
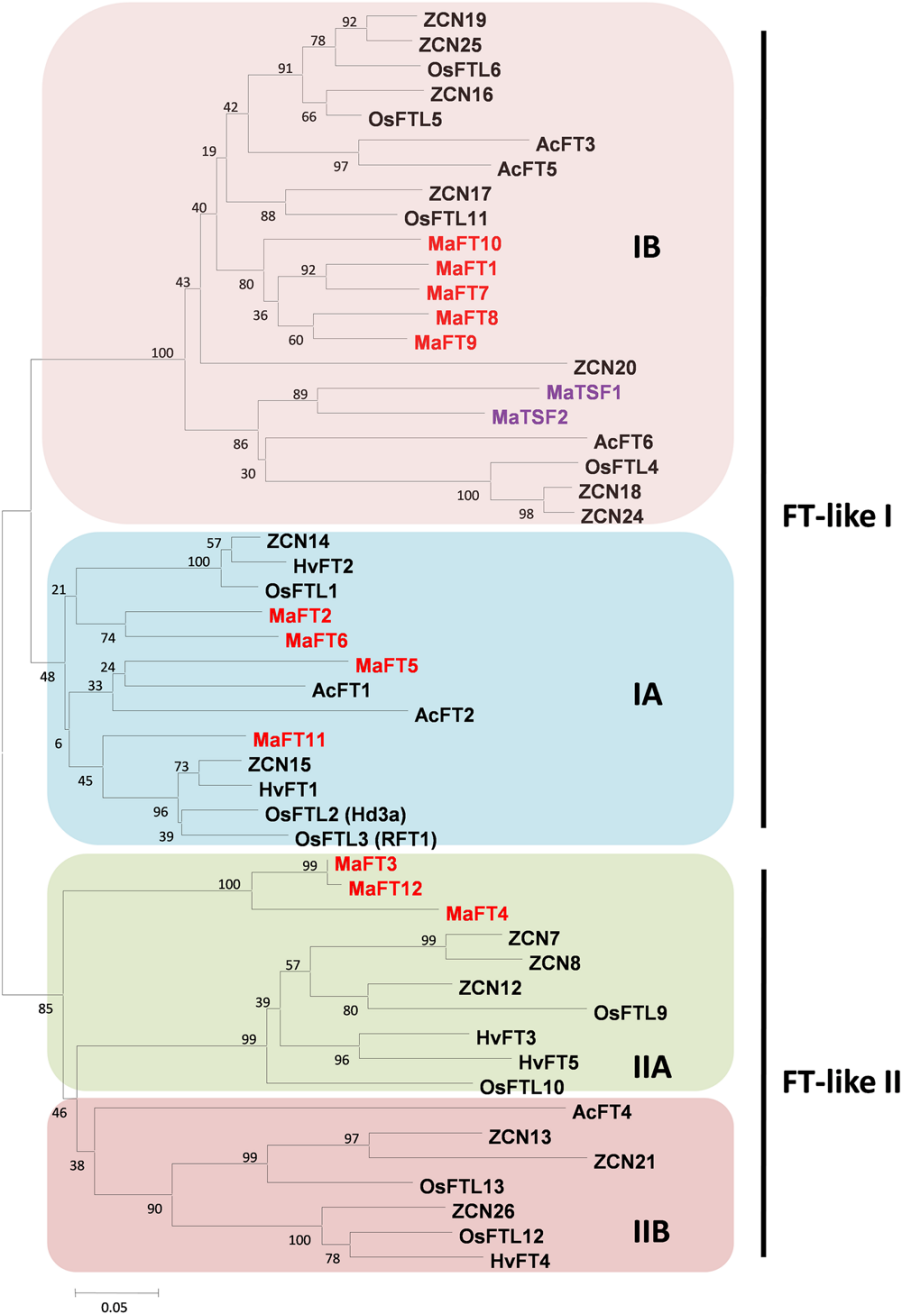
Phylogenetic analysis of banana FT/TSF-like family proteins with those from other monocots. The banana FT-like genes are shown in red, while TSF-like are in purple. Sequences were aligned with ClustalW and the tree was constructed with MEGA6.0 software using neighbour-joining analysis. The bootstrap values are shown in numbers in each branch for 1000 repetitions in percentage. The scale bar (0.05) reflects the amino acid substitution frequency as established by Poisson corrective distance model. Accession numbers are provided in Supplementary Tables S7 and S8.

In order to understand the relationship between the FT-like genes from banana and other monocots, a phylogenetic tree was constructed (Fig. 3). The various FT-like polypeptides clustered into four subgroups namely IA, IB, IIA and IIB as reported^24, 27^. Of these, subgroup IA which had functionally important FT proteins like Hd3aA and RFT1 (rice), HvFT1 and HvFT2 (barley), AcFT1 and AcFT2 (onion) contained MaFT2, MaFT5, MaFT6 and MaFT11. Subgroup IB contained the two TSF-like polypeptides MaTSF1 and MaTSF2 besides MaFT1 and MaFTs7-10 and also included Arabidopsis AtFT and AtTSF (Fig. S1). Interestingly, this sub-group with 87–93% similarity amongst the FTs, included all genes possessing the additional fourth intron. Sub-group IIA with ZCN8 as a functional FT included MaFT3, MaFT12 and MaFT4, with 92–99% similarities amongst themselves (Fig. 3, Supplementary Table S2). Subgroup IIB did not contain any banana FT/TSF like protein.

Amongst themselves, the MaFT polypeptides revealed more than 75% similarity (Supplementary Table S2). Of these, MaFT3 and MaFT12 (98.9% identity) differed in only two amino acids at positions 4 and 158 while MaTSF2 showed the lowest similarity (up to 56%) to other FT-like proteins. A comparison with functional FT proteins of other plants revealed MaFT11, MaFT7, MaFT5 and MaFT2 to be most similar to the functional AtFT, AtTSF and OsHD3 while MaFT3 and MaFT12 showed a greater similarity to ZCN8 (Supplementary Table S3).

**Figure 4:**
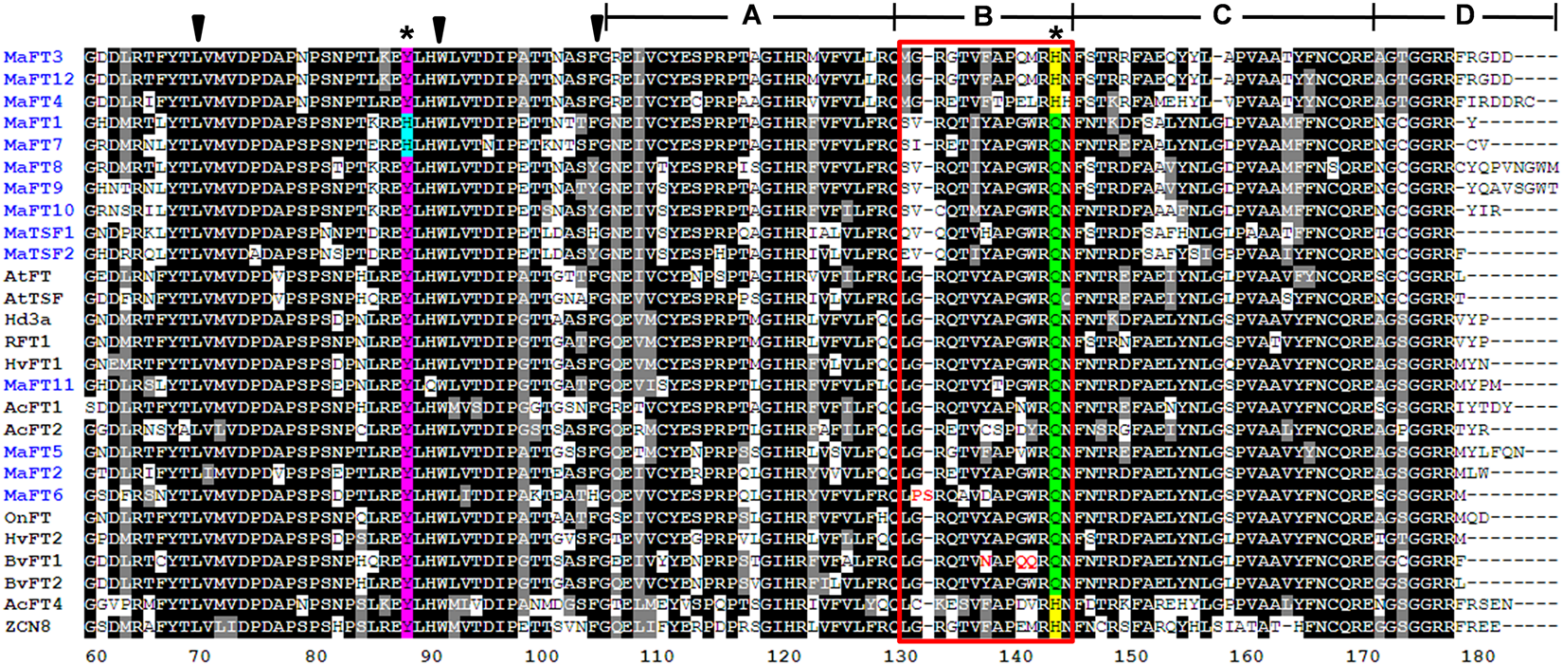
Alignments of partial deduced amino acid sequences of banana FT/TSF-like family proteins. The banana genes are highlighted in blue with functionally reported proteins in other plants, such as Arabidopsis, rice, barley, maize, orchid, sugarbeet and onion (accession numbers provided in materials and methods). Sequences were aligned using ClustalW and analyzed with BoxShade programme. Highly conserved amino acids are in black, grey or light depending on their level of identity (darker > grey > light). Triangles indicate the intron positions, which are conserved in plant PEBP family. Asterisks indicate the amino acid positions identified by Hanzawa *et al*.^41^ as being related to antagonistic functions of FT and TFL1. The segment B within the box corresponds to an external P loop which is a potential ligand-binding pocket in the FT/TFL1 family.

Amino acid alignment with other FTs revealed a few interesting changes despite overall conservation amongst the different FTs (Fig. 4). The crucial tyrosine and glutamine residues at positions equivalent to Tyr85 and Glu140 in AtFT^41^ remained as such in MaFT2, MaFT5, MaFT6, MaFT8-MaFT11, MaTSF1 and MaTSF2. The spacing between these two residues (55 amino acids) was also conserved in all except MaFT6. In MaFT3, MaFT4 and MaFT12 the glutamine (equivalent to Glu140 in AtFT) was replaced by histidine although this appears to be a functionally acceptable replacement as reported in the maize FT homologue ZCN8^42, 43^ and soybean GmFT5a^44^. Strikingly however, the crucial tyrosine (equivalent to Tyr85 in AtFT) was replaced by histidine in MaFT1 and MaFT7, a change that has been shown to be a characteristic of TFL1-like proteins^41^. The highly conserved B segment, crucial to FT function, differed most in MaFT6 in being 15 residues long (compared to 14 in FTs) and with a few substitutions within this normally invariant region. These included replacement of a conserved G (equivalent to G129 in AtFT) by a P, followed by an additional S that was absent in others. In addition, an A in place of T (T132 in AtFT) and a D in place of a conserved Y/F (Y134 in AtFT) was also noted. All members belonging to group IB, (MaFT1, MaFT7-10, MaTSF1 and MaTSF2) had certain prominent changes that included L to K/E/D at position 82, A to E at position 95, a G to V/I at 129, an E to A at position 149 and S/T to C at 170 (equivalent to positions in AtFT).

### Several FT/TSF-like genes are activated prior to flowering initiation in two differently flowering banana genotypes Grand Nain and Hill Banana

**Figure 5:**
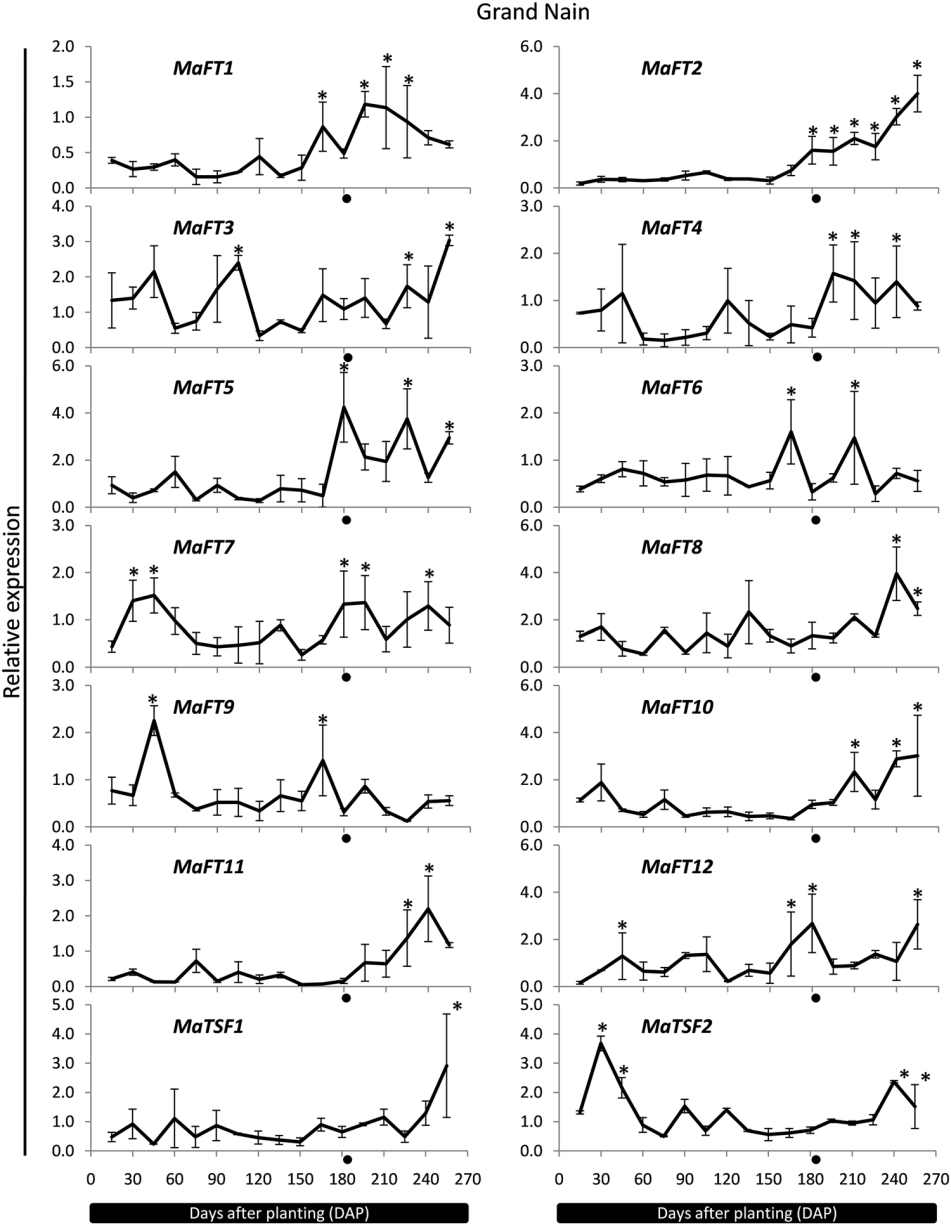
Time-course expression patterns of banana *FT*/*TSF* genes during development in Grand Nain by qRT-PCR. The *RPS2* gene of each is used as reference gene. The transition from vegetative to reproductive stage is shown by an asterisk. The values are means of two biological (each containing a pool of leaves of five plants) and three technical replicates (±SE). Analysis was performed using the Tukey-Kramer multiple comparison test by GraphPad software. The asterisks (*) indicate significant differences (P < 0.05).

In order to get an insight into the flowering related function of the banana FT/TSF-like genes, temporal changes in expression of these genes was studied at different stages of plant development. In Grand Nain, the first visible signs of floral differentiation are seen after day 180 within the pseudostem^45^ but not before day 165. The transcript levels of *MaFT1*, *MaFT2* and *MaFT5* (as well as *MaFT7* and to a lesser extent *MaFT12*), began to increase just prior to this stage from day 150 onwards with levels going significantly up (~2–6 folds) relative to their expression from 120–150 days (Fig. 5). There was little expression before this period except for *MaFT7* and *MaFT12* indicating that the rise in transcript level was likely related to flowering initiation. The expression of *MaFT4*, *MaFT10* and to some extent *MaTSF1* also began to increase prior to flowering. In addition, a minor peak at 120–135 days was seen for *MaFT1*, *MaFT4*, *MaFT7* and *MaFT12*. *MaFT11* increased just after flowering initiation from day 180 onwards with peak expression at 240–270 days that coincided with fruit development. Considerable expression during flower/fruit development was also seen for *MaFT2*, *MaFT3*, *MaFT5*, *MaFT8*, *MaFT10* and *MaTSF1*. Transcript levels of *MaFT6* and *MaFT9* showed a peak between 150 and 180 days but transcript levels reduced at the time of flowering. Expression of *MaFT3*, *MaFT8* and *MaTSF2* showed a fluctuating pattern with reduced expression during flowering indicating roles other than flowering. At least eight genes (*MaFT3*, *MaFT4*, *MaFT7*, *MaFT9*, *MaFT10*, *MaFT12*, *MaTSF1* and *MaTSF2*) also showed a prominent transcript peak early in development between 30 and 60 days. For all genes, only the completely spliced form was detected or was the most dominant form at all stages of development (data not shown).

**Figure 6:**
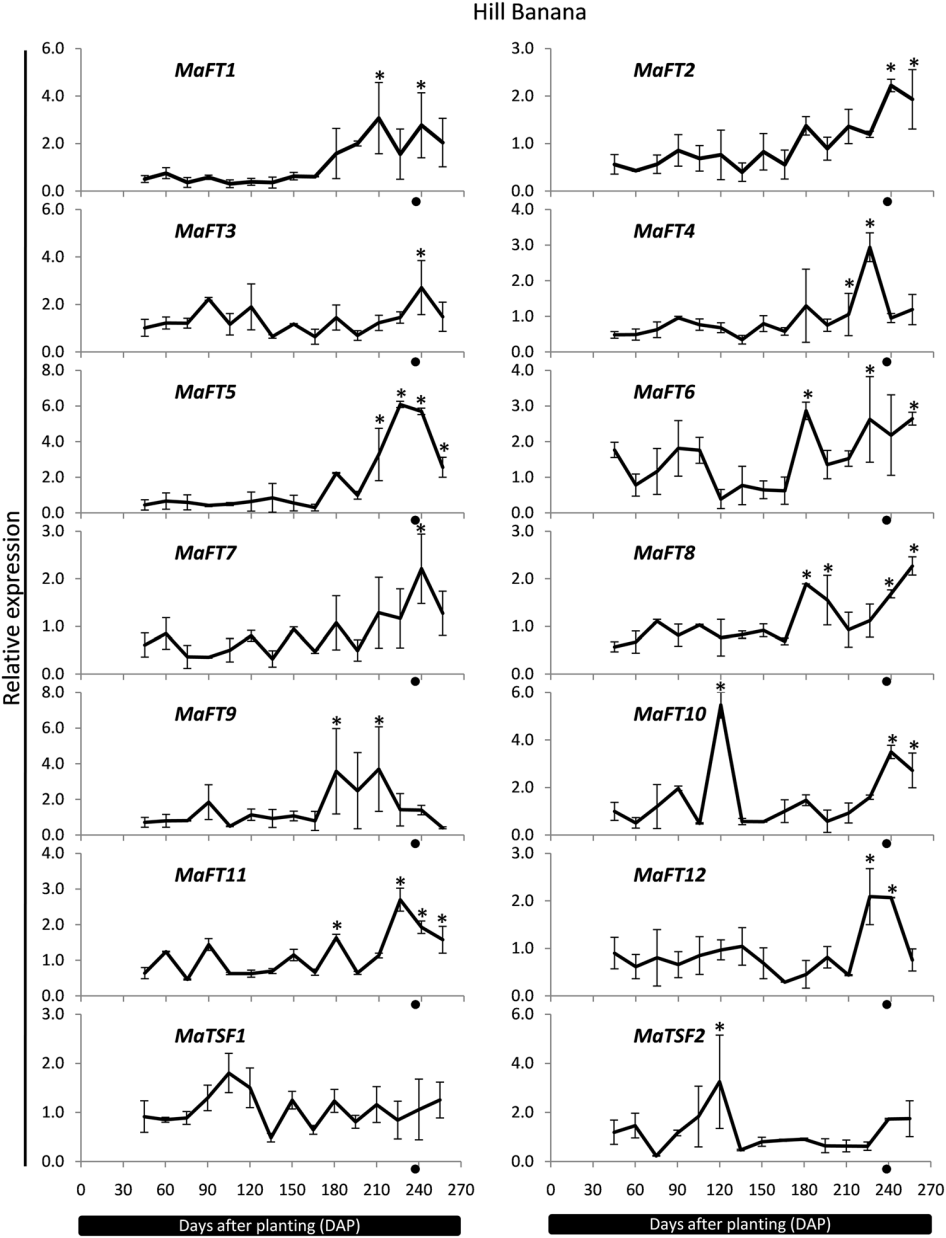
Time-course expression patterns of banana *FT*/*TSF* genes during development in Hill banana by qRT-PCR. The *RPS2* gene of each is used as reference gene. The transition from vegetative to reproductive stage is shown by a black circle. The values are means of two biological (each containing a pool of leaves of five plants) and three technical replicates (±SE). Analysis was performed using the Tukey-Kramer multiple comparison test by GraphPad software. The asterisks (*) indicate significant differences (P < 0.05).

The expression of the FT/TSF family was also studied in another genotype, Hill banana (AAB), due to differences in its height and time of flowering. Compared to Grand Nain which had a height of about 1.95 m in the fields (1.8–2 m as per Robinson and Sauco, 2010)^46^, Hill banana had an average height of 3 m (3–3.5 m, Rao, 2014)^47^. The appearance of the inflorescence was delayed by about 45–60 days compared to Grand Nain. As shown in Fig. 6, the expression of most banana FT/TSF genes largely mirrored that of Grand Nain with expression of *MaFT1*, *MaFT2*, *MaFT5* and MaFT12 showing a significant flowering related increase as in Grand Nain. High transcript levels at flowering were also seen in *MaFT3*, *MaFT6*, *MaFT7*, *MaFTs10–12*. In case of *MaFT1*, *MaFT4*, *MaFT6*, *MaFT7* and *MaFT9*, there was a delay in peak accumulation of transcript by about 15–30 days compared to Grand Nain while that of *MaFT2* and *MaFT5* was similar to Grand Nain. Compared to Grand Nain, both *MaFT8* and *MaF10* showed an additional peak at 180 and 120 days while expression of *MaTSF1* was quite different from Grand Nain.

### Most FT/TSF genes show day time and night peaks of mRNA accumulation

**Figure 7:**
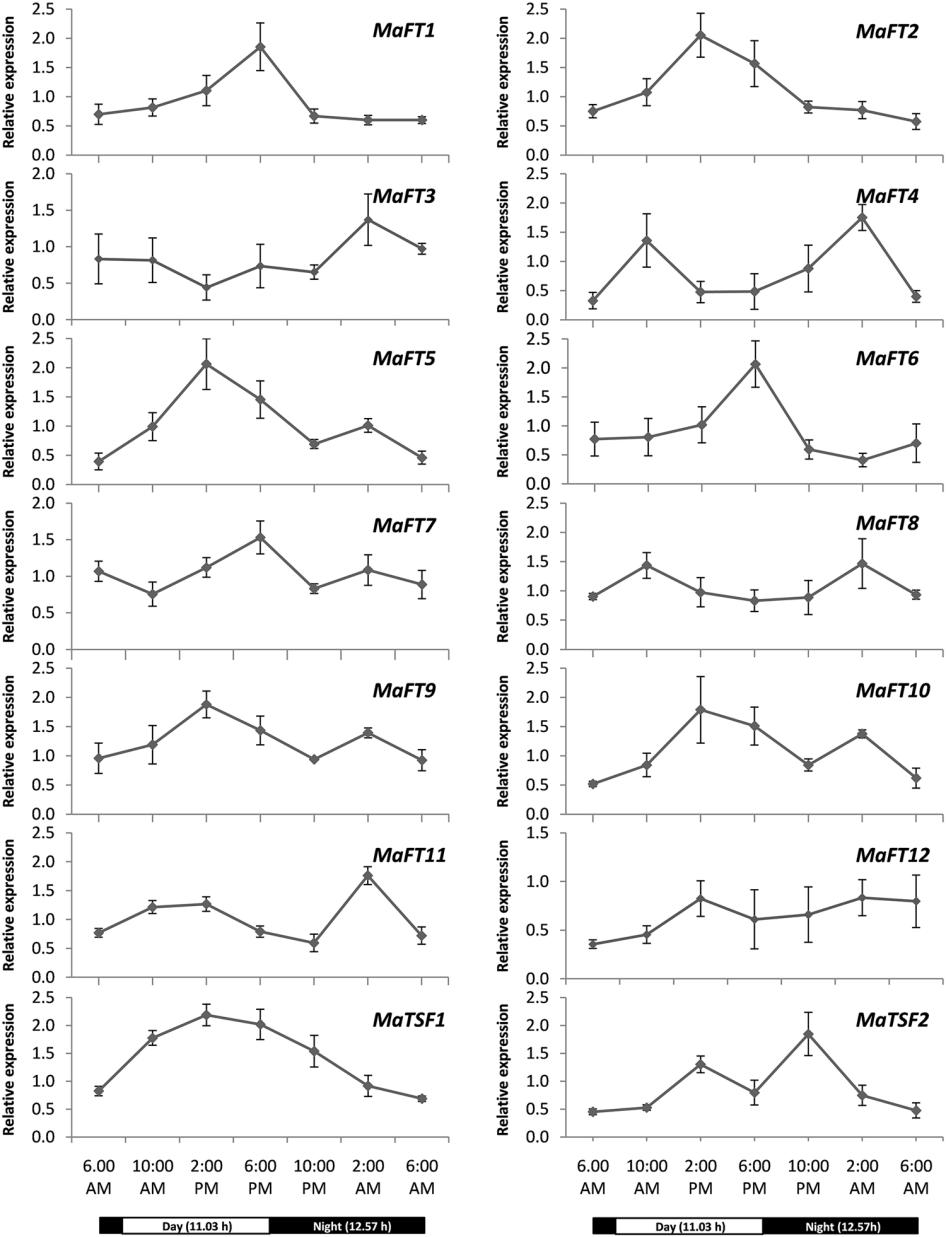
Diurnal expression patterns of *FT*/*TSF*-like genes of banana by qRT-PCR. Black and white bars at the bottom represent day and night periods. The relative expression was normalized against *RPS2*. The values are means of three biological and three technical replicates (±SE).

The FT-like genes in many plants show a peak of expression at a specific time such as just before evenings as in Arabidopsis *AtFT* under long days^2^, at sunrise as in Poplar *FT2* ^48^ or just before dawn as in rice *Hd3a* under short days^49^. In view of this, the diurnal expression patterns of the different banana FT/TSF family members were investigated over a 24 hour period. As shown in Fig. 7, all the FT/TSF-like genes except *MaFT3* and *MaFT11* showed a diurnal or semidiurnal expression pattern. *MaFT2*, *MaFT5*, *MaFT9*, *MaFT10*, *MaFT12*, *MaTSF1* and *MaTSF2* showed a distinct peak around 2 pm. Of these, *MaTSF1* expressed to high levels throughout the light period extending up to midnight. *MaFT1*, *MaFT6* and *MaFT7* peaked at dusk (around 6 pm) while *MaFT4* and *MaFT8* peaked in the morning at 10 am. Interestingly 9 of the 14 genes also showed a night time peak. For *MaFT3*, *MaFT4*, *MaFT8*, *MaFT11* and *MaTSF2* the night time peak was either greater than or as prominent as the day time peak while for *MaFT5*, *MaFT9* and *MaFT10* it was less prominent. In all cases except *MaTSF2*, the night peak was observed at 2 am, four hours before dawn.

### Banana FT/TSF-like genes are also expressed in tissues other than leaves

**Figure 8:**
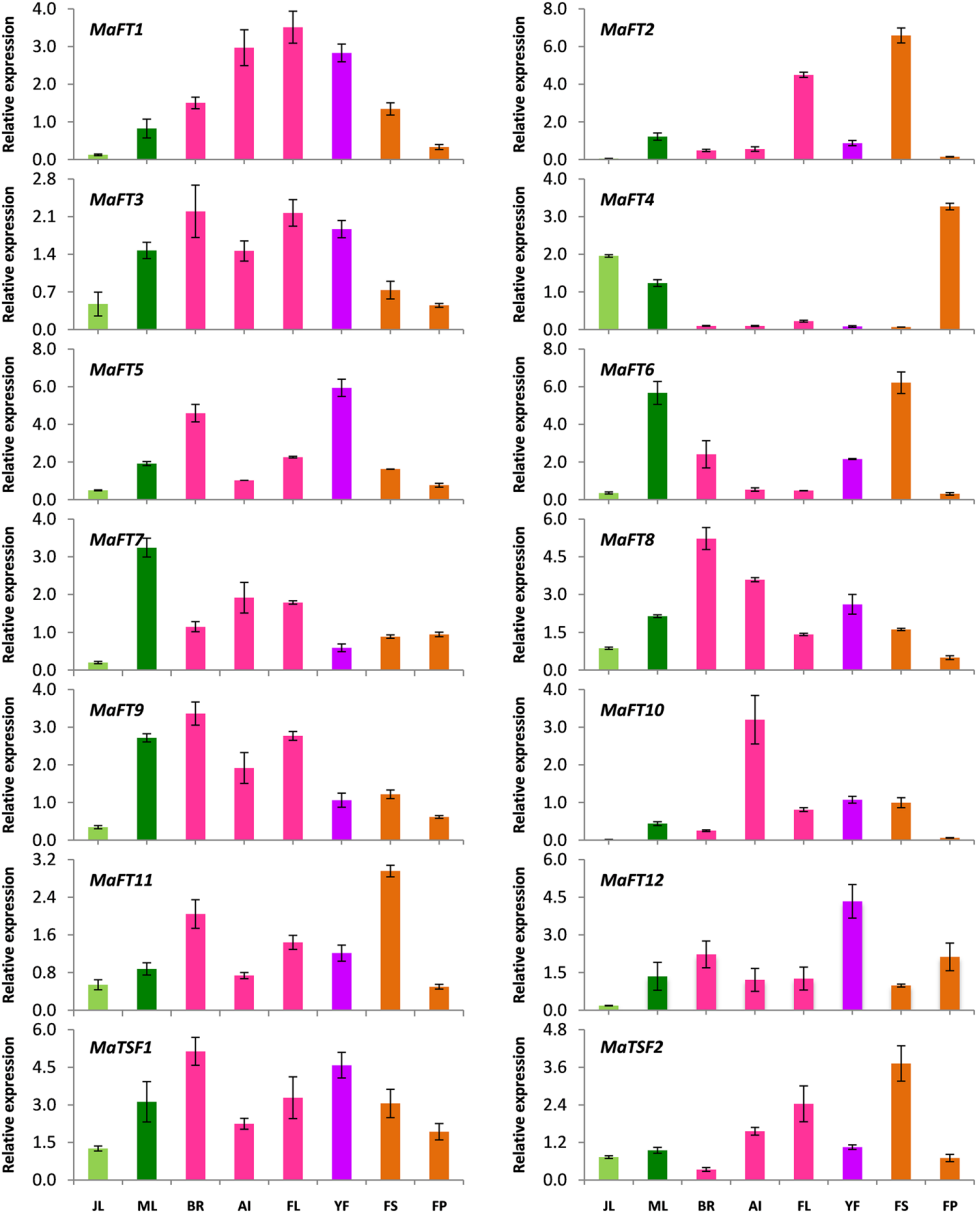
Tissue and organ-specific expression of banana *FT*/*TSF*-like genes. The relative expression was normalized against *RPS2*. The values are means of three biological and three technical replicates (±SE). JL-juvenile leaf, ML-mature leaf, BR-bract, AI- apical inflorescence, FL- mature flower, YF-young flower, FS-fruit skin, FP-fruit pulp.

Although FT genes have been associated with flowering time control in most plants, recent reports show their involvement in several other processes^38^. Hence, the expression patterns of the 14 FT/TSF like genes was investigated in eight different vegetative and reproductive tissues of banana. As shown (Fig. 8), most members of the FT family had a broad and overlapping expression pattern covering both vegetative and reproductive tissues. *MaFT1*, *MaFT3*, *MaFT9*, *MaFT11* and *MaTSF1* were expressed in most tissues but with reduced expression in fruit pulp. *MaFT6*, *MaFT7* and *MaFT9* were most prominently expressed in the mature leaves. Inflorescence tissues such as bracts, apical inflorescence and flowers showed the expression of *MaFT1*, *MaFT2* (mainly flowers), *MaFT3*, *MaFT5*, *MaFT7*, *MaFT8*, *MaFT9*, *MaFT11*, *MaFT12* and *MaTSF1* with most expressing highly in bracts. *MaFT10* was expressed predominantly in the apical inflorescence with very little expression in other tissues. Interestingly, most FT genes were also expressed considerably in fruit tissues such as the fruit skin (*MaFT2*, *MaFT6*, *MaFT11* and *MaTSF2*) and fruit pulp (*MaFT4* and *MaFT12*). *MaFT4* seemed to be excluded from floral tissues, bracts and fruit skin. These observations suggested that FT/TSF genes might have specific roles in other aspects of development in different tissues.

### Nine of the 14 *FT*/*TSF* genes can functionally complement the mutant defect in Arabidopsis *ft*-*10* mutant

**Figure 9:**
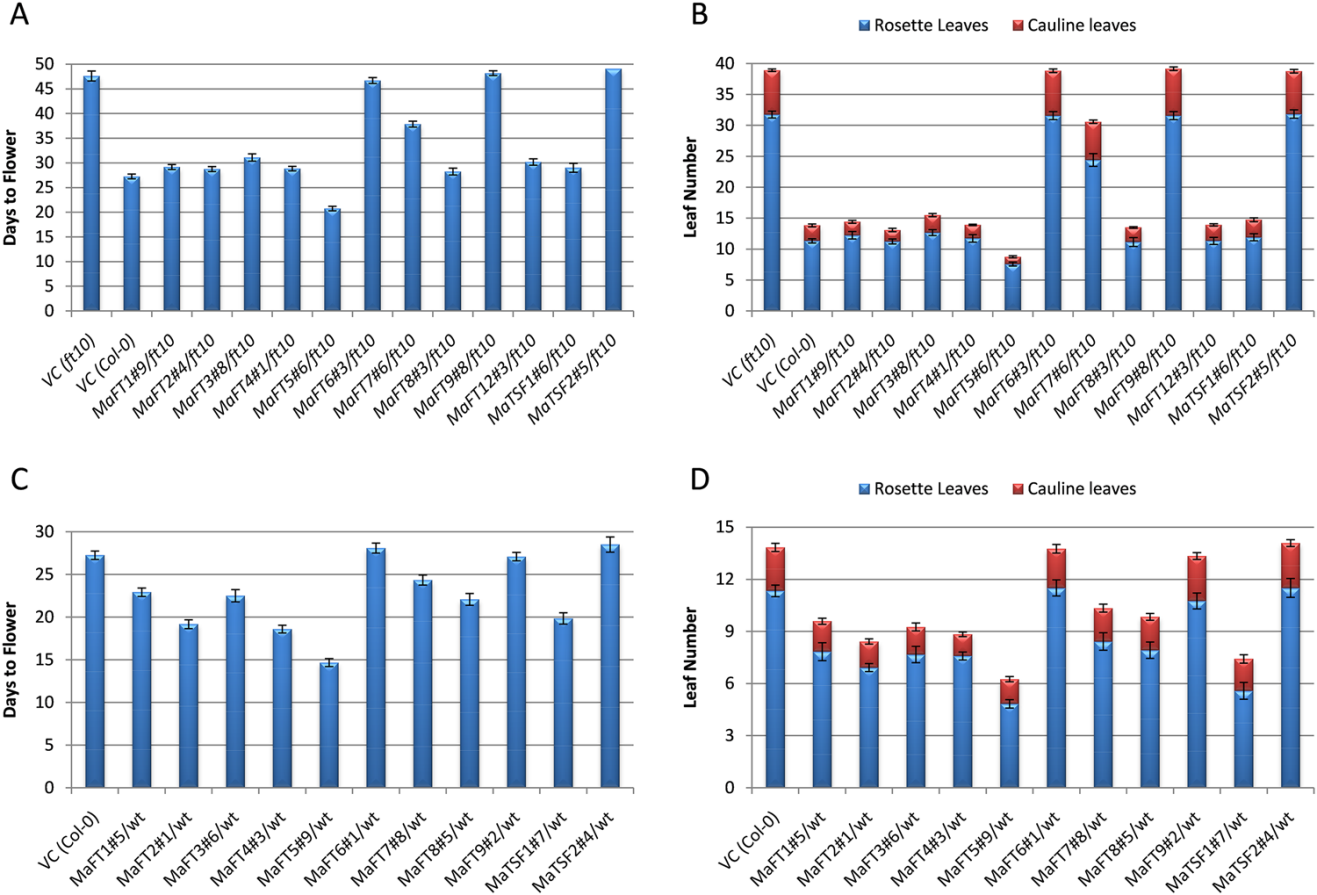
Expression of the banana *FT*/*TSF*-like genes in the *ft*-*10* and Col-0 background of *Arabidopsis*. Various banana *FT*/*TSF*-like genes were expressed under the CaMV35S promoter in the *ft*-*10* mutant background (**A** and **B**) and the Col-0 background (**C** and **D**) of Arabidopsis. Days to flower (**A**,**C**) were counted as number of days taken to bolt under long day conditions (16 h light, 8 h dark). Leaf number (**B**,**D**) was calculated by combining total rosette leaves and cauline leaves on primary inflorescence. For each line, a minimum of 12 plants were selected and data shown is average of independent lines ± SE for each gene.

The functionality of the banana FT/TSF-like genes was next tested by investigating their ability to suppress the flowering defect in the Arabidopsis *ft*-*10* mutant upon expression under the CaMV35S promoter. Flowering in the *ft*-*10* mutant is considerably delayed and occurs in 46–48 days (32–33 leaves) compared 26–27 days (11–12 leaves) in Col-0 under long days. Of the 14 FT/TSF like genes, twelve could be tested in the *ft*-*10* mutant. As shown in Fig. 9(A and B) and Supplementary Fig. S2, eight of these namely, *MaFT1*-5, *MaFT8*, *MaFT12* and *MaTSF1* could completely suppress the late flowering phenotype of the *ft*-*10* mutant. Most independent lines expressing these genes flowered in 27–30 days with 10–12 leaves like the Col-0 control plants compared to 46–47 days (33 leaves) in the *ft*-*10* mutant. *MaFT5* expressers flowered even earlier than the Col-0 controls with 7–8 leaves (20–21 days). *MaFT7* was also able to suppress the *ft*-*10* mutant phenotype, albeit weakly, with *MaFT7* expressers flowering in 36–37 days (23–25 leaves) compared to *ft*-*10*. In contrast, *MaFT6*, *MaFT9* and *MaTSF2* did not suppress the *ft*-*10* mutant defect and flowered in 46–48 days (32–33 leaves) indicating that these genes were unable to function as FTs at least in Arabidopsis.

The ability of the FT/TSF-like genes to impart early flowering by over-expression in the Col-0 background was also tested. As shown in Fig. 9(C and D) and Supplementary Fig. S3, over-expression of eight of the eleven genes tested under the CaMV35S promoter namely, *MaFT1*-*5*, *MaFT8*, *MaFT12* and *MaTSF1* led to early flowering between 18–23 days (6–8 leaves) with *MaFT5* expressing plants flowering even earlier in 14–15 days (4–5 leaves). *MaFT7* expressers flowered earlier than Col-0 but later than the others in 24 days with 8–9 leaves. *MaFT6*, *MaFT9* and *MaTSF2* had no effect on flowering while *MaFT10* and *MaFT11* could not be tested.

**Figure 10:**
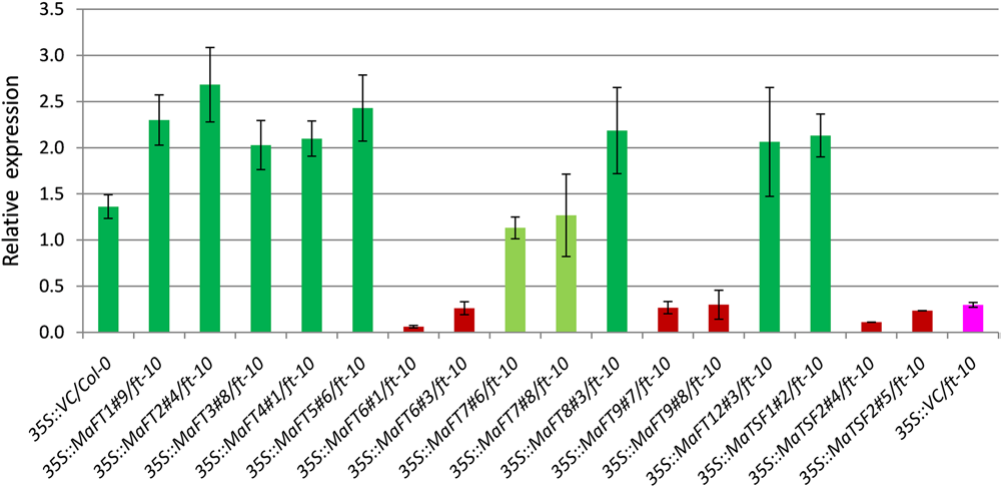
Detection of Arabidopsis *AP1* expression by qRT-PCR in transgenic Arabidopsis lines ectopically expressing banana *MaFT*/*TSF* genes. The *SAND* (At2g28390) gene of Arabidopsis was used as reference gene.

An analysis of the expression of the floral identity gene *AP1* which is a target of FT and necessary to initiate flowering was carried out in Arabidopsis lines expressing the different banana *FT* genes in the *ft*-*10* background. A clear up-regulation of *AP1* was seen in all lines (Fig. 10) except those expressing *MaFT6*, *MaFT9* and *MaTSF2* in spite of the genes being expressed in transgenic plants (Fig. S4). Relatively lower transcript levels were seen in lines expressing *MaFT7* that matched the partial restoration phenotype of *MaFT7*.

Collectively these studies show that at least nine of the 14 FT/TSF like genes namely *MaFT1*-*MaFT5*, *MaFT7*, *MaFT8*, *MaFT12* and *MaTSF1* could functionally interact with the flowering components even in Arabidopsis suggesting a structure that is conserved with AtFT and capable of being recognized by the FT/FD complex of Arabidopsis.

## Discussion

### Banana genome contains 14 FT/TSF-like genes

Banana is one of the most important day neutral fruit crops forming the staple food of many African and Asian countries. In the tropics, banana flowers in 7–9 months and completes its first fruit harvest in about 12 months regardless of the season. Although it is grown in most places in Central India under micro-irrigated conditions, its long life cycle makes it susceptible to water stress under the hot summer conditions or to cold stress in North Indian winter conditions. Hence manipulation of its life cycle for reduction of flowering time is desirable. Yet, in spite of its economic importance, there have been no detailed studies on the PEBP family genes that play a key role in flowering time regulation.

The current studies in the triploid (AAA) variety of dessert banana Grand Nain, aided by the recently completed AA double haploid genome sequence^40^ show the presence of 12 *FT*-like and two *TSF*-like genes. This is a large number compared to dicots but appears to be a feature of monocots exemplified by the presence 13 in rice^25^, 15 in maize^24^, 13 in sorghum^50^, 9 in barley^25^, at least 6 in onion^27^ and 6 each in *Brachypodium* and wheat^28^. Although varying at the genomic level due to intronic variation, exonic structures are largely conserved with exons 2 (62 nt) and 3 (41 nt) being invariant in size. This feature of the exonic structure has been reported across plants in both monocots such as maize^24^, rice^25, 51^, barley^26^, sugarcane^52^ and in eudicots, Arabidopsis^5, 6^, apple^23^, tomato^20^, poplar^21^, Medicago^53^ etc. Interestingly, *MaFT1*, *MaFT8*, *MaFT9* and *MaTSF2* displayed four introns instead of three that have so far been observed in all plants except the dicot *Gypsophila paniculata* ^39^. The additional intron interrupted the last exon exactly after 217 bp in all the four genes just as observed in *G*. *paniculata* ^39^ which is surprising considering the evolutionary distance between the two. Further studies in other plant *FT*s may shed light on how widespread the fourth intron is and on the origin of the additional intron/exon in a gene family that has an otherwise highly conserved exonic structure.

### Banana has more than one *FT*/*TSF*-like flowering inducer genes

The 14 FT/TSF-like proteins show greater than 75% similarity in their amino acid sequence amongst themselves and more than 60% with AtFT, AtTSF1 and OsHd3a suggesting high conservation. Indeed, functionality studies carried out in Arabidopsis show that eight of the twelve genes studied, namely *MaFT1*-*MaFT5*, *MaFT8*, *MaFT12*, and *MaTSF1* could efficiently suppress the delayed flowering phenotype of the *ft*-*10* mutant while *MaFT7* weakly suppressed the defect. These genes also imparted early flowering in the Col-0 ecotype upon over-expression indicating the ability of the encoded proteins to recognize and interact with components of the flowering machinery even in a distantly related plant like Arabidopsis. The results thus highlight the remarkable conservation in FT structure/function in plants as diverse as banana and Arabidopsis. Interestingly, *MaFT6*, *MaFT9* and *MaTSF2* failed to suppress the *ft*-*10* phenotype in spite of high similarity to other FTs suggesting that certain amino acid differences within these proteins might prevent their recognition by the flowering machinery components in plants. An exhaustive analysis of amino acids and determinants necessary for FT function has already been performed through several studies^22, 41, 54, 55^. The differences due to genetic diversity in banana FTs provides additional interesting study material to reveal further intricacies in the FT structure and function. As an example, MaFT9 and MaTSF2 in spite of being very similar to MaFT8 and MaTSF1 respectively (Figs 3 and 4) are unable to suppress the flowering defect in *ft*-*10* or induce early flowering in Col-0 upon over-expression. This implies that certain residues that differ between the pairs MaFT8/MaFT9 and MaTSF1/MaTSF2 may be important in deciding FT functionality at least in the context of the Arabidopsis flowering machinery. An important finding of our studies is that the Y to H substitution in both MaFT1 and MaFT7 at the position equivalent to amino acid 85 in AtFT, is not deleterious to their function as FT unlike that reported by Hanzawa *et al*.^41^. This is evident from the ability of both these proteins to suppress the flowering defect in *ft*-*10* (albeit weakly in case of MaFT7) as well as induce early flowering through over-expression in Col-0 despite this change. This suggests that both FTs can tolerate this change so long as there are other simultaneous changes (as yet unidentified) within the proteins that suppress the effect of the Y to H change and allow them to continue to function as FTs. At least the crucial amino acids that supposedly interact with Y through hydrogen bonding^55^, do not appear to differ between MaFT1/MaFT7 and other FTs indicating other subtle changes within these two FTs. So far, MaFT1 and MaFT7 represent the only two proteins in literature with the Y to H substitution at position 85 (equivalent to Arabidopsis AtFT) where early flowering has been reported. The differences in the abilities of these two proteins to suppress the *ft*-*10* defect might be due to subtle differences within their sequences. The ability of MaFT5 to induce flowering earlier than any other banana gene in both the Col-0 background and the mutant *ft*-*10* background also highlights the fact that differences in regions other than the B segment may influence FT function. Simultaneously, the importance of the conserved B segment, which is invariant with 14 residues in FTs^54^, is also apparent from the inability of *MaFT6* to promote flowering. MaFT6 contains a 15 amino acid B segment with crucial changes in the conserved residues. B segments longer than 14 residues and varied in amino acid composition are features of plant TFL1-like genes. However, unlike TFL1s, *MaFT6* did not delay flowering over that of control upon over-expression in Arabidopsis. Nevertheless, one cannot rule out *MaFT6* as a flowering gene since minor differences in the FT proteins (that prevent interactions in Arabidopsis as seen for MaFT6, MaFT9 and MaTSF2) may be associated with complementing differences in the interacting partners such as FD thus still allowing an efficient complex to be formed in banana.

Although a large number of the FT/TSF-like genes in banana can function like AtFT, only a few can be actually correlated with the transition from vegetative to reproductive growth that occurs between 170–190 days in Grand Nain and a little later in Hill banana. As expected from such a function, transcript levels of *MaFT1*, *MaFT2* and *MaFT5* begin increasing between 150–180 days, going up by 2–6 fold compared to their levels at 120–150 days not only in Grand Nain but also in the delayed flowering Hill banana. Five others namely *MaFT4*, *MaFT7*, *MaFT10*, *MaFT12* and *MaTSF1* also showed an increase in transcript accumulation between 150/165–180 (*MaFT7*, *MaFT10* and *MaFT12*) and 120–150 (*MaFT4* and *MaFT7*) days indicating a possible flowering related function. The expression of most of these was largely conserved even in Hill banana, and delayed slightly in tune with the delay in appearance of the inflorescence. Since the Hill banana is taller than Grand Nain at least some delay in the visible inflorescence may be due to the greater distance that has to be covered by the growing inflorescence in Hill banana rather than entirely due to a delay in development of the inflorescence meristem. This might explain the lesser delay in expression pattern of the FT genes in Hill banana compared to the actual delay observed between Grand Nain and Hill banana. The coordinated increase in transcript levels of seven of the 14 FT/TSF genes prior to flowering may provide a robust means for initiation of flowering. However, identifying the relative contribution of individual genes to flowering will require further studies through genetic manipulation which is somewhat difficult in banana. Whether the diurnal cycling of the transcripts with day and night time peaks might also be linked to flowering is difficult to say as of now.

Other genes like *MaFT7*, *MaFT9*, *MaFT3* and *MaTSF2* that show a peak in transcript levels soon after transplanting may have additional functions. This period coincides with the formation of ratoons. In plants like onion and potato, where bulbing and tuberization is observed, the expression of FT genes like *AcFT1* and *AcFT4* in onion and *StSP3A* in potato has been correlated with bulb^27^ and tuber formation^37^ respectively. An interesting possibility could be a similar function for these genes in formation of suckers or ratoons. Many genes are also transcribed in tissues like fruit pulp, peel, flowers and bracts at levels higher than those in mature leaves and persist long after flowering initiation. The expression in these tissues is similar to reports in apple^23^, maize^24^ and Medicago^53^ and clearly suggests a role that must be different from flowering and possibly more diverse in monocots given the large expansion of the family. For instance, in maize only 8 of the 15 FT like genes were transcribed in leaves and only *ZCN8* has so far been associated with flowering suggesting other roles for the other members. In contrast to maize, where tissue/stage specific splicing differences were observed, most of the banana FT/TSF like members showed only the completely spliced form in all tissues studied (data not shown) suggesting that regulation by alternative splicing is unlikely in banana. Knocking out these genes, although time consuming and difficult due to the triploid nature of dessert banana and its inability to set seeds, may provide a clue to their function in these tissues and is currently in progress. Nevertheless, the structural similarity amongst the different proteins (that act in flowering and non-flowering functions) suggests that they might have similar interacting surfaces for other partners (possibly other members of the bZIP and 14-3-3 family) even when they are active in non-flowering related functions.

Taken together, sequence analysis and gene expression studies in Grand Nain and Hill banana coupled with functional analysis identify at least three MaFTs (*MaFT1*, *MaFT2*, *MaFT5*) and possibly *MaFT7* as candidates for flowering promotion with other FTs possibly aiding the process. These observations provide a base for further studies for confirming their roles in flowering for future manipulation of flowering time in banana.

## Methods

### Plant material and growth conditions

All experiments were carried out on the banana cultivars, ‘Grand Nain’ (*Musa acuminata* L., a dwarf Cavendish banana of AAA genotype) and ‘Hill Banana’ (*Musa acuminata* L. ecotype Virupakshi, a tall banana of AAB genotype). The plants were grown under field conditions (Supplementary Fig. S5) at the R and D Farm of Jain Irrigation Systems Limited, Jalgaon, Maharashtra, India.

*Arabidopsis thaliana* ecotype Columbia (Col-0) and the *ft*-*10* mutant^56^ defective in the *AtFT* gene, were used for functional characterization of the FT/TSF-like genes from banana. Col-0, *ft*-*10* and transgenic Arabidopsis lines were grown on soilrite mix (TC grade, Keltech Energies, Bangalore) in growth rooms under fluorescent light (120–150 µmol m^−2^ s^−1^) at constant temperature of 21 °C under long day (LD) conditions of 16 hours light and 8 hours dark.

### Genomic DNA isolation and Genome walking library preparation

Genomic DNA was isolated from leaves by the modified plant DNA mini-preparation method^57^. The genome walking library was prepared with isolated genomic DNA using the Genome Walker Universal kit (Clontech Laboratories, Inc. USA) as per manufacturer’s instructions.

### RNA extraction, cDNA and SMART RACE library preparation

RNA was isolated from mature leaves of tissue cultured plants at the hardening stage, six and nine month old field growing plants, developing apical inflorescence (inside the pseudostem of seven month old plants) and various flower parts as described^58^ with minor modifications and purified using RNeasy Plant Mini Kit (Qiagen, Germany). A mixed cDNA library was prepared from this RNA.

For tissue specific studies, total RNA was extracted from juvenile leaves of hardening stage plants, mature leaves, bracts, apical inflorescence, mature flowers (from 8 month old flower bearing plants), mature fruit skin and pulp (from 9–10 month old fruit bearing plants) as described by Chaurasia *et al*.^45^.

For developmental expression studies, leaf samples were collected every 15 days (Grand Nain) and 45 days (Hill Banana) after planting for a period up to 255 days. For each sample, the fully mature, expanded, newly emerged leaf was chosen. Samples were collected from ten independent field plants between 1 to 2 PM and two pools of five independent leaves were prepared.

For diurnal analysis of gene expression, leaf samples (fully mature, expanded, newly emerged leaves) were collected every four hours from 150 day old field growing plants as described above in triplicates.

First strand cDNA was synthesized using the iScript cDNA synthesis kit (Bio-Rad) along with the 3′AP (5′ AAGCAGTGGTATCAACGCAGAGTACTTTTTTTTTT TTTTTTTT3′; Invitrogen) as per manufacturer’s instructions. Separate SMART cDNA libraries (5′ and 3′ SMART) were prepared using SMART RACE cDNA Amplification Kit (Clontech Laboratories, Inc. USA). A pooled mix of total RNA from various tissues was used for library preparation. Sequences of all the primers used are provided in Supplementary Table S6 or stated otherwise.

### Cloning of banana FT and TSF-like genes

Partial genomic sequences of three FT-like genes designated as *MaFLT1* (Ac No DQ153045, 211 bp), *MaFLT2* (Ac No DQ153047, 219 bp), and *MaFLT3* (Ac No DQ153048, 217 bp), were obtained from the GenBank database of Musa AAB group cultivar ‘Horn plantain’. Gene specific primers (Supplementary Table S6) based on these partial sequences were used on SMART cDNA libraries and Genome Walker libraries (Clontech Laboratories, Inc. USA) to obtain the flanking sequences for cDNA (inclusive of 5′ and 3′ UTRs) as well as genomic DNA. Once the composite sequence was available, primers were designed to amplify the complete open reading frames of *MaFT1* (*MaFLT1*), *MaFT2* (*MaFLT2*) and *MaFT3* (*MaFLT3*) from a mix of cDNA libraries prepared from RNA isolated from leaves of various stages and reproductive tissues.

Additionally, degenerate primers MaFT-A9-F, MaFT-A19-F and MaFT-B6-R were designed based on an alignment of several FT family sequences from different plants using COnsensus DEgenerate Hybrid Oligonucleotide Primers (iCODEHOP v1.1; http://dbmi-icode-01.dbmi.pitt.edu/i-codehop-context/) interactive programme. PCR amplification for isolation of additional members of the banana FT family was carried out using the TrueStart HotStart Taq DNA Polymerase (Fermentas, EU) as follows: initial denaturation of 95 °C for 5 min, followed by 36 cycles of 30 s at 95 °C, 30 s at 60 °C, and 2 min at 72 °C with a final extension of 10 min at 72 °C. Genome walking and SMART-RACE PCRs were performed using Advantage 2 PCR Kit (Clontech Laboratories, Inc. USA) as per the manufacturer’s conditions using gene specific (GSP) and adapter primers provided by the company. The fragments were cloned in pTZ57R/T using the InsTAclone PCR Cloning Kit (Fermentas, EU) and sequenced.

MaFT4 was obtained as a partial genomic sequence of 745 bp using degenerate primers FT-A19-F and FT-B6-R. The 5′/3′ flanking sequences were obtained using two forward and reverse internal intron-specific primers (Table S6) in combination with adapter primers AP1 and AP2.

During the course of this work the entire banana genome sequence (DH-Pahang variety, AA genome), a double haploid of *Musa acuminata* genotype became available^40^. The database (http://banana-genome.cirad.fr) was searched using sequences of the four FT-like genes obtained. The search yielded twelve genes of the FT family that showed similarity to either rice *Hd3a*-like or other *FT*-like genes. These included the four *FT*-like genes *MaFT1*, *MaFT2*, *MaFT3* and *MaFT4* identified in the lab. In addition, two TSF-like genes (*MaTSF1* and *MaTSF2*) were also obtained. Based on the sequences, gene specific primers (Table S6) were designed for isolation of the ten additional genes designated as *MaFT5*-*12* (FT family) and *MaTSF1* and *MaTSF2* (TSF family). Full-length ORFs of *MaFT5*, *MaFT6*, *MaFT7*, *MaFT11* and *MaTSF1* were amplified by Phusion high-fidelity DNA polymerase (Thermo Fisher Scientific, Finland) using a pooled cDNA library while 5′ and 3′ UTRs were obtained by SMART-RACE PCR.

Primer combinations for *MaFT8*, *MaFT9*, *MaFT10* and *MaTSF2* failed to amplify the corresponding fragments from pooled cDNAs due to a mistake in database in annotation of the termination codon. Hence genomic DNA was used as a template to obtain the DNA fragments for these genes. Two reverse primers (3′ UTR-R1 and 0R1) were designed and used to obtain the full length cDNAs of *MaFT8*, *MaFT9* and *MaTSF2*. Sequence analysis revealed the presence of a fourth intron in each of these genes that changed the termination codon from that predicted in the database.

Comparison of the banana FTs with FT proteins in other plants was carried out by an alignment using ClustalW and analyzed using the programme BoxShade. The aligned sequences included Arabidopsis (*AtFT*, NP_176726; *AtTSF*, NP_193770), rice (*OsHd3a*, AB052942; *RFT1*, AB062676), barley (*HvFT1*, DQ100327; *HvFT2*, DQ297407), maize (*ZCN8*, EU241900), orchid (*OnFT*, EU583502), sugarbeet (*BvFT1*, HM448910; *BvFT2*, HM448912) and onion (*AcFT1*, KC485348; *AcFT2*, KC485349; *AcFT4*, KC485351). Phylogenetic analysis of banana FT/TSF-like family proteins with FT-like proteins from monocots (accession numbers in Supplementary Table S7) was carried out by MEGA6.0 software using neighbour-joining analysis.

DNA sequences of each banana FT/TSF gene from this article can be found at the NCBI database (http://www.ncbi.nlm.nih.gov/) under the accession numbers provided in Supplementary Table S8.

### qRT-PCR Analysis

The transcript level of each gene was quantified by quantitative real time PCR using gene specific primers (Supplementary Table S1), generated by Primer3 software (version 0.4.0) using the SsoFast EvaGreen Supermix (Bio-Rad) on a CFX96 Real-Time PCR Detection System (Bio-Rad, USA). The thermocycler was programmed as follows: 2 min at 50 °C, 95 °C for 10 min, followed by 40 cycles of 15 s at 95 °C, 10 s at 57 °C, and 15 s at 72 °C. The data was analyzed by the CFX Manager Software programme (Bio-Rad). Relative gene expression was normalized against expression of ribosomal protein S2 (*RPS2*; HQ853246)^45, 59^ and calculated from the cycle time (Ct value) using the ΔΔCt method. The amplicon size of each primer pair was confirmed by RT-PCR and by sequencing (Xcelris Labs, Gujarat, India) and the efficiency of primers checked on a dilution series of mixed cDNA. Statistical analysis for expression was performed using the Tukey-Kramer multiple comparison test by GraphPad software and significant differences (P < 0.05) were marked.

### Plasmid Construction and Arabidopsis transformation

Coding sequences of different *FT*-like genes were amplified with gene specific forward (0F) and reverse (0R) primers that included the initiation and termination codons respectively (Table S6) using Phusion high-fidelity DNA polymerase on pooled cDNA synthesized from various tissues. The amplified fragments were initially cloned in pTZ57R/T, sequenced to confirm the absence of mutations followed by cloning in the binary vector pBI121. Arabidopsis transformation of the wild type Col-0 ecotype and the *ft*-*10* mutant was performed by floral dip method^60^ using the GV3101 strain of *Agrobacterium*. At least four independent transformants (and a maximum of up to eight) were chosen for detailed studies of each gene. Homozygous T3 progeny (12 plants for each independent line) were grown and monitored for flowering time, leaf number at bolting, number of cauline leaves in the Col-0 and *ft*-*10* mutant backgrounds.

## Electronic supplementary material

Supplementary Information
