# Supplementary material for: Flowering time in banana (Musa spp.), a day neutral plant, is controlled by at least three FLOWERING LOCUS T homologues
Source: Sci Rep. 2017 Jul 19;7:5935. doi: 10.1038/s41598-017-06118-x (PMC5517511; doi:10.1038/s41598-017-06118-x)
Supplement: Supplementary file 1 — Supplementary Information [file 41598_2017_6118_MOESM1_ESM.pdf]

**Flowering time in banana (*Musa spp.*), a day neutral plant, is controlled by at least four putative *FLOWERING LOCUS T* homologues**

Akhilesh K. Chaurasia, Hemant B. Patil, Bal Krishna, V.R. Subramaniam, Prafullachandra V. Sane and Aniruddha P. Sane

## Supplementary Tables and figures

**Supplementary Table S1. The details and coordinates of 14 *FT*/*TSF*-like genes of banana taken from banana genome database** (http://banana-genome.cirad.fr).

| Banana Genome Annotation (Gene/Locus) | Chr | Start | End | Strand | Query Coverage | Name given in this study | Amino Acid Residues (n) |
|---|---|---|---|---|---|---|---|
| GSMUA_Achr9T07900_001~ Protein HEADING DATE 3A~ HD3A~ | chr9 | 5067756 | 5068866 | - | 99.91 | ***MaFT1*** | 172 |
| GSMUA_Achr10T21780_001~ Protein HEADING DATE 3A~ HD3A~ | chr10 | 27215563 | 27216458 | + | 99.89 | ***MaFT2*** | 174 |
| GSMUA_Achr5T06600_001~ Protein HEADING DATE 3A~ HD3A~ | chr5 | 4856092 | 4856954 | + | 99.88 | ***MaFT3*** | 175 |
| GSMUA_Achr2T19360_001~ Protein FLOWERING LOCUS T~ FT~ | chr2 | 19463406 | 19464372 | - | 99.90 | ***MaFT4*** | 177 |
| GSMUA_Achr4T08540_001~ Protein HEADING DATE 3A~ HD3A~ | chr4 | 6274754 | 6275889 | - | 99.91 | ***MaFT5*** | 179 |
| GSMUA_Achr2T19310_001~ Protein HEADING DATE 3A~ HD3A~ | chr2 | 19414161 | 19416534 | - | 99.96 | ***MaFT6*** | 175 |
| GSMUA_Achr2T05870_001~ Protein FLOWERING LOCUS T~ FT~ | chr2 | 10806987 | 10808797 | + | 99.94 | ***MaFT7*** | 173 |
| GSMUA_Achr10T16460_001~ Protein HEADING DATE 3A~ HD3A~ | chr10 | 23980518 | 23982407 | + | 99.95 | ***MaFT8*** | 180 |
| GSMUA_Achr3T02730_001~ Protein FLOWERING LOCUS T~ FT~ | chr3 | 1776128 | 1779521 | - | 99.97 | ***MaFT9*** | 178 |
| GSMUA_Achr2T20900_001~ Protein HEADING DATE 3A~ HD3A~ | chr2 | 20579763 | 20581731 | + | 99.95 | ***MaFT10*** | 180 |
| GSMUA_Achr10T21760_001~ Prot;ein HEADING DATE 3A~ HD3A~ | chr10 | 27212259 | 27213048 | + | 99.87 | ***MaFT11*** | 177 |
| GSMUA_Achr5T06420_001~ Protein FLOWERING LOCUS T~ FT~ | chr5 | 4708329 | 4709190 | - | 99.88 | ***MaFT12*** | 175 |
| GSMUA_Achr1T17050_001~ Protein TWIN SISTER OF FT~ TSF~ | chr1 | 12729700 | 12733006 | + | 99.91 | ***MaTSF1*** | 173 |
| GSMUA_Achr3T21510_001~ Protein TWIN SISTER OF FT~ TSF~ | Chr3 | 22452853 | 22457385 | - | 99.98 | ***MaTSF2*** | 174 |

**Supplementary Table S2. Identity (upper triangle in bold) and similarity (lower triangle) percentage of putative banana FT/TSF-like proteins.** The results were generated by MatGat software with default parameters.

| | **MaFT1** | **MaFT2** | **MaFT3** | **MaFT4** | **MaFT5** | **MaFT6** | **MaFT7** | **MaFT8** | **MaFT9** | **MaFT10** | **MaFT11** | **MaFT12** | **MaTSF1** | **MaTSF2** |
|---|---|---|---|---|---|---|---|---|---|---|---|---|---|---|
| **MaFT1** | – | **67.8** | **62.5** | **59.0** | **63.7** | **67.4** | **87.9** | **79.4** | **82.1** | **81.6** | **68.4** | **61.9** | **68.4** | **72.4** |
| **MaFT2** | 79.9 | – | **68.8** | **66.9** | **77.7** | **81.9** | **69.5** | **63.9** | **94.8** | **66.7** | **78.0** | **68.2** | **60.8** | **63.6** |
| **MaFT3** | 78.3 | 81.7 | – | **82.5** | **68.2** | **63.7** | **64.8** | **62.8** | **62.0** | **61.4** | **65.2** | **98.9** | **59.6** | **59.6** |
| **MaFT4** | 76.8 | 79.1 | 92.1 | – | **67.8** | **60.2** | **61.2** | **60.0** | **59.2** | **60.7** | **62.2** | **83.1** | **60.0** | **56.1** |
| **MaFT5** | 79.3 | 82.1 | 86.0 | 82.1 | – | **71.1** | **65.4** | **62.6** | **60.8** | **62.6** | **78.2** | **68.7** | **58.1** | **59.2** |
| **MaFT6** | 79.4 | 89.7 | 79.4 | 75.1 | 81.0 | – | **66.5** | **61.7** | **63.2** | **65.0** | **75.8** | **63.1** | **61.7** | **61.1** |
| **MaFT7** | 93.6 | 80.5 | 78.9 | 78.5 | 79.3 | 79.4 | – | **80.0** | **80.4** | **82.2** | **68.4** | **64.2** | **68.0** | **70.3** |
| **MaFT8** | 87.2 | 77.8 | 76.1 | 76.7 | 78.9 | 77.2 | 89.4 | – | **85.6** | **78.9** | **64.8** | **62.2** | **66.5** | **67.0** |
| **MaFT9** | 88.8 | 78.7 | 77.0 | 75.8 | 78.2 | 78.7 | 89.9 | 91.7 | – | **84.4** | **66.9** | **61.7** | **68.5** | **69.1** |
| **MaFT10** | 89.1 | 79.9 | 76.6 | 76.8 | 78.2 | 77.7 | 91.4 | 88.3 | 92.1 | – | **68.9** | **60.8** | **71.1** | **72.2** |
| **MaFT11** | 79.1 | 84.7 | 80.8 | 79.1 | 87.7 | 82.5 | 81.4 | 81.1 | 82.0 | 81.4 | – | **64.6** | **59.3** | **62.7** |
| **MaFT12** | 78.3 | 81.7 | 99.4 | 92.1 | 86.0 | 79.4 | 78.9 | 76.1 | 77.0 | 76.6 | 80.8 | – | **59.6** | **59.0** |
| **MaTSF1** | 83.8 | 78.7 | 77.1 | 76.3 | 74.9 | 80.0 | 86.7 | 81.1 | 83.1 | 83.9 | 77.4 | 77.7 | – | **78.7** |
| **MaTSF2** | 85.1 | 79.3 | 78.3 | 76.3 | 77.7 | 77.7 | 86.8 | 81.7 | 83.1 | 84.5 | 78.0 | 78.9 | 91.4 | – |

**Supplementary Table S3. Identity and similarity percentage of putative banana FT/TSF-like proteins with Arabidopsis (AtFT), rice (Hd3a) and maize (ZCN8) functional proteins.**

| | Identity / Similarity (%) | | | |
|---|---|---|---|---|
| **Gene** | **AtFT** | **AtTSF** | **Hd3a** | **ZCN8** |
| **MaFT1** | 68.8 / 81.1 | 66.9 / 79.4 | 68.2 / 79.9 | 55.1 / 71.4 |
| **MaFT2** | 71.2 / 82.9 | 70.6 / 81.7 | 76.0 / 83.8 | 61.4 / 76.6 |
| **MaFT3** | 63.7 / 81.7 | 64.8 / 80.0 | 64.6 / 81.6 | 60.2 / 82.9 |
| **MaFT4** | 63.0 / 79.1 | 61.9 / 80.2 | 61.2 / 78.2 | 56.7 / 79.1 |
| **MaFT5** | 71.7 / 83.8 | 70.6 / 81.6 | 76.9 / 87.7 | 60.9 / 75.4 |
| **MaFT6** | 69.3 / 81.7 | 68.8 / 77.1 | 73.1 / 83.2 | 57.3 / 71.4 |
| **MaFT7** | 71.0 / 85.1 | 69.3 / 82.9 | 67.0 / 80.4 | 57.4 / 72.6 |
| **MaFT8** | 63.4 / 78.9 | 64.5 / 78.9 | 66.5 / 80.0 | 54.1 / 70.6 |
| **MaFT9** | 67.0 / 80.9 | 65.9 / 80.9 | 65.2 / 81.6 | 53.3 / 71.3 |
| **MaFT10** | 68.9 / 81.7 | 66.7 / 81.7 | 67.6 / 81.0 | 52.8 / 70.9 |
| **MaFT11** | 71.3 / 85.3 | 70.2 / 81.4 | 78.9 / 91.6 | 58.8 / 72.9 |
| **MaFT12** | 64.2 / 81.7 | 64.2 / 80.0 | 64.1 / 81.6 | 59.7 / 82.9 |
| **MaTSF1** | 63.4 / 78.9 | 65.1 / 79.4 | 59.2 / 76.5 | 52.5 / 71.4 |
| **MaTSF2** | 64.0 / 79.4 | 66.3 / 77.7 | 62.0 / 77.1 | 51.4 / 70.3 |

**Supplementary Table S4:** Flowering time analysis of individual plants over-expressing various banana *FT*/*TSF*-like genes in an *ft-10* mutant background. Values are means ± SE calculated using Tukey-Kramer multiple comparison test. Asterisks indicate a statistically significant difference between plants in the same column (*P<0.05, ** P<0.01, ***P<0.001 respectively).

| Line | LD conditions (16 h light/8 h dark period) | | | | |
|---|---|---|---|---|---|
| | **No. of Plants** | **Days to Flower** | **Rosette leaves** | **Cauline leaves** | **Total leaves** |
| *ft-10* | 12 | 48.50±1.0 | 32.17±0.6 | 7.34±0.3 | 39.50±0.8 |
| VC(*ft-10*) | 12 | 47.58±1.0 | 31.75±0.6 | 7.17±0.2 | 38.91±0.7 |
| Wt (Col) | 12 | 27.84±0.5*** | 11.08±0.3*** | 2.58±0.2*** | 13.67±0.4*** |
| VC/Wt (Col) | 12 | 27.25±0.5*** | 11.33±0.3*** | 2.50±0.2*** | 13.84±0.5*** |
| **35S::*MaFT1*/*ft-10*** | | | | | |
| #6 | 12 | 30.33±0.6*** | 12.75±0.6*** | 2.33±0.1*** | 15.08±0.5*** |
| #9 | 12 | 29.17±0.7*** | 12.25±0.6*** | 2.16±0.2*** | 14.42±0.7*** |
| #10 | 12 | 30.50±0.7*** | 12.60±0.7*** | 2.08±0.2*** | 14.70±0.7*** |
| #11 | 12 | 29.83±0.8*** | 12.33±0.6*** | 2.25±0.2*** | 14.58±0.8*** |
| **35S::*MaFT2*/*ft-10*** | | | | | |
| #3 | 12 | 27.17±0.6*** | 12.50±0.5*** | 1.75±0.2*** | 14.25±0.5*** |
| #4 | 12 | 28.75±1.0*** | 11.25±0.4*** | 1.84±0.3*** | 13.08±0.5*** |
| #5 | 12 | 28.08±0.9*** | 11.67±0.6*** | 2.41±0.2*** | 14.08±0.6*** |
| #7 | 12 | 27.30±0.9*** | 11.30±0.6*** | 2.17±0.2*** | 13.50±0.7*** |
| #8 | 12 | 29.10±0.8*** | 12.40±0.6*** | 2.58±0.2*** | 15.00±0.6*** |
| **35S::*MaFT3*/ *ft-10*** | | | | | |
| #8 | 12 | 31.08±0.8*** | 12.67±0.5*** | 2.84±0.3*** | 15.50±0.4*** |
| #9 | 12 | 30.84±0.9*** | 12.17±0.5*** | 2.08±0.3*** | 14.25±0.6*** |
| #10 | 12 | 29.67±0.7*** | 12.25±0.5*** | 2.75±0.3*** | 15.00±0.5*** |
| #12 | 12 | 30.42±0.5*** | 13.08±0.5*** | 2.42±0.3*** | 15.50±0.6*** |
| **35S::*MaFT4*/ *ft-10*** | | | | | |
| #1 | 12 | 28.83±0.2*** | 11.75±0.6*** | 2.16±0.1*** | 13.92±0.6*** |
| #2 | 12 | 32.58±0.7*** | 15.50±0.7*** | 2.84±0.2*** | 18.34±0.9*** |
| #4 | 12 | 28.17±0.4*** | 11.67±0.6*** | 2.16±0.2*** | 13.84±0.6*** |
| #5 | 12 | 27.34±0.8*** | 11.42±0.5*** | 2.50±0.2*** | 13.92±0.6*** |
| #6 | 12 | 26.84±0.7*** | 11.00±0.5*** | 1.75±0.2*** | 12.75±0.6*** |
| #10 | 12 | 27.67±0.7*** | 11.67±0.5*** | 1.91±0.2*** | 13.58±0.6*** |
| #14 | 12 | 26.84±0.7*** | 10.42±0.5*** | 1.75±0.2*** | 12.17±0.5*** |
| #15 | 12 | 27.17±0.7*** | 11.42±0.5*** | 2.34±0.1*** | 13.75±0.6*** |
| **35S::*MaFT5*/ *ft-10*** | | | | | |
| #4 | 12 | 20.75±0.7*** | 7.58±0.3*** | 1.16±0.2*** | 8.75±0.5*** |
| #5 | 12 | 19.92±0.5*** | 6.75±0.4*** | 1.25±0.1*** | 8.00±0.4*** |
| #6 | 12 | 21.08±0.6*** | 8.00±0.4*** | 1.00±0.2*** | 9.00±0.5*** |
| #7 | 12 | 25.17±0.7*** | 10.00±0.8*** | 1.41±0.2*** | 11.42±0.7*** |
| #10 | 12 | 24.25±0.4*** | 10.08±0.4*** | 1.34±0.1*** | 11.42±0.6*** |
| #11 | 12 | 24.50±0.4*** | 9.50±0.4*** | 1.58±0.1*** | 11.08±0.5*** |
| #12 | 12 | 20.92±0.3*** | 6.91±0.1*** | 1.08±0.2*** | 8.00±0.2*** |
| #17 | 12 | 26.08±0.6*** | 11.58±0.5*** | 1.75±0.2*** | 13.34±0.6*** |
| **35S::*MaFT6*/ *ft-10*** | | | | | |
| #1 | 12 | 45.58±1.0 | 30.34±0.7 | 7.08±0.4 | 37.42±0.8 |
| #2 | 12 | 45.00±1.3 | 30.92±0.5 | 7.00±0.3 | 37.92±0.6 |
| #3 | 12 | 46.67±1.1 | 31.58±0.7 | 7.25±0.3 | 38.84±0.8 |
| #4 | 12 | 47.17±1.2 | 32.08±1.0 | 7.25±0.4 | 39.34±1.1 |
| #6 | 12 | 45.08±1.1 | 31.42±0.5 | 7.75±0.3 | 39.17±0.6 |
| #11 | 12 | 46.84±1.2 | 32.67±0.6 | 7.25±0.4 | 39.92±0.8 |
| #14 | 12 | 47.00±1.0 | 31.92±0.7 | 7.84±0.3 | 39.75±0.8 |
| #17 | 12 | 48.17±1.1 | 32.17±0.8 | 7.75±0.3 | 39.92±0.7 |

Table S4**: Continued**

| Line | LD conditions (16 h light/8 h dark period) | | | | |
|---|---|---|---|---|---|
| | **No. of Plants** | **Days to Flower** | **Rosette leaves** | **Cauline leaves** | **Total leaves** |
| **35S::*MaFT7/ ft-10*** | | | | | |
| #5 | 12 | 37.83±1.0*** | 24.42±1.0*** | 6.16±0.3 | 30.58±1.3*** |
| #6 | 12 | 38.42±1.2*** | 26.00±0.8*** | 6.58±0.4 | 32.58±1.1*** |
| #8 | 12 | 38.17±0.9*** | 23.42±0.8*** | 5.58±0.2** | 29.00±0.9*** |
| #9 | 12 | 36.25±0.8*** | 20.25±0.8*** | 4.66±0.2*** | 24.92±0.7*** |
| **35S::*MaFT8/ ft-10*** | | | | | |
| #1 | 12 | 28.25±0.6*** | 10.92±0.5*** | 2.42±0.2*** | 13.33±0.5*** |
| #2 | 12 | 28.75±0.4*** | 11.17±0.6*** | 2.50±0.2*** | 13.67±0.7*** |
| #3 | 12 | 28.25±0.5*** | 11.17±0.7*** | 2.33±0.1*** | 13.50±0.8*** |
| #4 | 12 | 27.20±0.5*** | 11.40±0.5*** | 2.42±0.2*** | 13.80±0.5*** |
| #5 | 12 | 28.67±0.6*** | 11.25±0.6*** | 2.50±0.2*** | 13.75±0.6*** |
| #6 | 12 | 27.08±0.5*** | 11.17±0.5*** | 2.50±0.2*** | 13.67±0.5*** |
| #7 | 12 | 27.83±0.4*** | 10.92±0.4*** | 2.58±0.1*** | 13.50±0.6*** |
| #8 | 12 | 27.10±0.5*** | 11.20±0.5*** | 2.42±0.2*** | 13.58±0.5*** |
| **35S::*MaFT9/ ft-10*** | | | | | |
| #1 | 12 | 47.33±0.8 | 32.92±0.9 | 7.17±0.4 | 40.08±1.0 |
| #2 | 12 | 48.42±1.0 | 33.08±0.8 | 7.25±0.3 | 40.33±0.8 |
| #3 | 12 | 46.42±1.1 | 30.83±1.0 | 7.25±0.3 | 38.08±1.1 |
| #4 | 12 | 46.17±0.7 | 31.67±0.9 | 7.58±0.2 | 39.25±0.8 |
| #5 | 12 | 45.50±1.2 | 31.42±0.7 | 7.08±0.4 | 38.50±0.9 |
| #6 | 12 | 47.92±0.7 | 31.50±0.9 | 7.16±0.3 | 38.67±1.1 |
| #7 | 12 | 48.08±0.9 | 32.42±0.7 | 7.50±0.3 | 39.92±0.6 |
| #8 | 12 | 48.17±1.0 | 31.58±0.6 | 7.58±0.3 | 39.17±0.6 |
| **35S::*MaFT12/ ft-10*** | | | | | |
| #1 | 12 | 31.17±0.8*** | 13.00±0.6*** | 2.17±0.3*** | 15.17±0.6*** |
| #2 | 12 | 31.50±0.7*** | 12.08±0.6*** | 2.25±0.3*** | 14.34±0.6*** |
| #3 | 12 | 30.17±0.7*** | 11.34±0.6*** | 2.58±0.2*** | 13.92±0.7*** |
| #5 | 12 | 29.34±0.7*** | 12.25±0.5*** | 2.92±0.3*** | 15.17±0.6*** |
| #6 | 12 | 29.92±0.5*** | 12.58±0.5*** | 2.84±0.3*** | 15.42±0.5*** |
| #7 | 12 | 31.08±1.0 | 13.00±0.9 | 6.84±0.2 | 37.92±1.0 |
| #8 | 12 | 30.25±0.7*** | 12.67±0.6*** | 2.75±0.4*** | 15.42±0.5*** |
| **35S::*MaTSF1/ ft-10*** | | | | | |
| #1 | 12 | 26.67±0.8*** | 12.34±0.5*** | 2.17±0.3*** | 14.50±0.6*** |
| #2 | 12 | 27.17±0.9*** | 12.08±0.6*** | 2.25±0.3*** | 14.34±0.6*** |
| #6 | 12 | 28.50±0.6*** | 11.34±0.6*** | 2.58±0.2*** | 13.92±0.7*** |
| #9 | 12 | 28.58±0.8*** | 12.08±1.6*** | 2.75±0.3*** | 14.84±0.5*** |
| #13 | 12 | 29.00±0.6*** | 11.92±0.6*** | 2.84±0.3*** | 14.75±0.6*** |
| #18 | 12 | 29.83±0.6*** | 11.75±0.6*** | 2.84±0.3*** | 14.58±0.4*** |
| **35S::*MaTSF2/ ft-10*** | | | | | |
| #1 | 12 | 48.34±1.1 | 32.25±0.7 | 6.17±0.3 | 38.42±0.8 |
| #2 | 12 | 48.58±1.2 | 31.25±0.7 | 6.25±0.3 | 37.50±0.7 |
| #3 | 12 | 47.67±0.9 | 31.00±0.7 | 6.58±0.2 | 37.58±0.7 |
| #4 | 12 | 47.42±1.3 | 32.08±0.9 | 6.17±0.4 | 38.25±1.1 |
| #5 | 12 | 49.08±0.7 | 31.83±0.7 | 6.92±0.3 | 38.75±0.8 |
| #8 | 12 | 48.25±0.8 | 31.75±0.7 | 6.83±0.3 | 38.58±0.7 |
| #11 | 12 | 47.67±1.5 | 30.83±1.0 | 7.08±0.2 | 37.92±1.0 |
| #18 | 12 | 48.17±0.9 | 32.50±0.7 | 6.75±0.4 | 39.25±0.6 |

**Supplementary Table S5:** Flowering time analysis of individual plants over-expressing various banana *FT*/*TSF*-like genes in a Col-0 background. Values are means ± SE calculated using Tukey-Kramer multiple comparison test. Asterisks indicate a statistically significant difference between plants in the same column (*P<0.05, ** P<0.01, ***P<0.001 respectively).

| Line | LD conditions (16 h light/8 h dark period) | | | | |
|---|---|---|---|---|---|
| | **No. of Plants** | **Days to Flower** | **Rosette leaves** | **Cauline leaves** | **Total leaves** |
| Wt (Col) | 12 | 27.84±0.5 | 11.08±0.3 | 2.58±0.2 | 13.67±0.4 |
| VC/Wt (Col) | 12 | 27.25±0.5 | 11.33±0.3 | 2.50±0.2 | 13.84±0.5 |
| **35S::*MaFT1*/wt** | | | | | |
| #5 | 12 | 22.92±0.5*** | 7.84±0.5*** | 1.75±0.2* | 9.58±0.5*** |
| #6 | 12 | 23.17±0.6*** | 8.08±0.5*** | 1.67±0.2 | 9.75±0.5*** |
| #7 | 12 | 22.67±0.6*** | 7.58±0.4*** | 1.84±0.2 | 9.42±0.4*** |
| #8 | 12 | 23.08±0.7*** | 8.17±0.5*** | 1.75±0.1 | 9.92±0.6*** |
| #9 | 12 | 22.75±0.7*** | 7.66±0.4*** | 1.75±0.2 | 9.42±0.5*** |
| **35S::*MaFT2*/wt** | | | | | |
| #1 | 12 | 19.17±0.5*** | 6.91±0.2*** | 1.50±0.2* | 8.41±0.3*** |
| #2 | 12 | 19.34±0.4*** | 6.84±0.4*** | 1.58±0.2* | 8.41±0.5*** |
| #3 | 12 | 19.84±0.6*** | 7.00±0.3*** | 1.41±0.1** | 8.41±0.4*** |
| #7 | 12 | 18.92±0.3*** | 6.67±0.4*** | 1.34±0.1*** | 8.00±0.4*** |
| #9 | 12 | 19.25±0.6*** | 6.50±0.4*** | 1.58±0.1* | 8.08±0.4*** |
| #10 | 12 | 18.84±0.4*** | 6.75±0.4*** | 1.34±0.1*** | 8.08±0.4*** |
| **35S::*MaFT3*/wt** | | | | | |
| #6 | 12 | 23.92±0.7** | 7.58±0.6*** | 1.75±0.2 | 9.33±0.6*** |
| #7 | 12 | 22.50±0.7*** | 7.66±0.5*** | 1.58±0.2* | 9.25±0.5*** |
| #8 | 12 | 23.25±0.8*** | 7.41±0.4*** | 1.83±0.2 | 9.08±0.5*** |
| #9 | 12 | 22.25±0.6*** | 7.25±0.4*** | 1.66±0.2 | 8.91±0.6*** |
| **35S::*MaFT4*/wt** | | | | | |
| #1 | 12 | 19.84±0.4*** | 7.41±0.3*** | 1.67±0.1 | 9.03±0.4*** |
| #2 | 12 | 17.92±0.3*** | 7.08±0.3*** | 1.84±0.1 | 8.91±0.3*** |
| #3 | 12 | 18.58±0.5*** | 7.58±0.2*** | 1.25±0.1*** | 8.84±0.2*** |
| #4 | 12 | 18.42±0.4*** | 6.84±0.4*** | 1.50±0.2* | 8.84±0.5*** |
| #9 | 12 | 18.50±0.3*** | 6.34±0.4*** | 1.67±0.2 | 8.00±0.4*** |
| #14 | 12 | 19.08±0.4*** | 6.34±0.4*** | 1.91±0.2 | 8.25±0.6*** |
| **35S::*MaFT5*/wt** | | | | | |
| #3 | 12 | 16.34±0.5*** | 5.41±0.3*** | 1.41±0.1** | 6.84±0.3*** |
| #6 | 12 | 15.67±0.4*** | 5.25±0.3*** | 1.34±0.1*** | 6.58±0.3*** |
| #7 | 12 | 14.42±0.4*** | 4.84±0.2*** | 1.25±0.1*** | 6.08±0.4*** |
| #8 | 12 | 15.75±0.5*** | 5.16±0.2*** | 1.58±0.1* | 6.75±0.3*** |
| #9 | 12 | 14.67±0.5*** | 4.84±0.2*** | 1.41±0.1*** | 6.25±0.3*** |
| #10 | 12 | 16.00±0.5*** | 5.34±0.3*** | 1.25±0.1*** | 6.58±0.4*** |
| **35S::*MaFT6*/wt** | | | | | |
| #1 | 12 | 28.08±0.6 | 11.50±0.5 | 2.25±0.3 | 13.75±0.6 |
| #2 | 12 | 27.17±0.5 | 10.84±0.4 | 2.67±0.2 | 13.50±0.4 |
| #3 | 12 | 28.75±0.6 | 11.17±0.5 | 2.75±0.1 | 13.92±0.5 |
| #6 | 12 | 29.08±0.7 | 11.50±0.5 | 2.67±0.1 | 14.17±0.5 |
| #9 | 12 | 27.75±0.8 | 11.50±0.5 | 2.25±0.1 | 13.75±0.5 |
| #10 | 12 | 29.17±0.5 | 11.58±0.5 | 2.83±0.1 | 14.42±0.5 |
| #12 | 12 | 28.75±0.8 | 11.42±0.5 | 2.41±0.1 | 13.83±0.5 |
| #13 | 12 | 28.42±0.6 | 12.00±0.5 | 2.33±0.1 | 14.33±0.5 |

Table S5**: Continued**

| Line | LD conditions (16 h light/8 h dark period) | | | | |
|---|---|---|---|---|---|
| | **No. of Plants** | **Days to Flower** | **Rosette leaves** | **Cauline leaves** | **Total leaves** |
| **35S::*MaFT7*/wt** | | | | | |
| #1 | 12 | 28.25±0.8 | 12.08±0.6 | 2.50±0.2 | 14.58±0.5 |
| #2 | 12 | 27.00±0.6 | 11.75±0.6 | 2.08±0.2 | 13.83±0.4 |
| #4 | 12 | 26.68±0.5 | 12.08±0.6 | 2.25±0.2 | 14.33±0.6 |
| #5 | 12 | 25.08±0.5 | 8.91±0.6* | 2.08±0.2 | 11.00±0.6** |
| #6 | 12 | 23.92±0.7** | 8.16±0.7*** | 1.91±0.3 | 10.08±0.7*** |
| #7 | 12 | 24.58±0.8 | 8.33±0.5** | 1.83±0.2 | 10.17±0.5*** |
| #8 | 12 | 24.33±0.6* | 8.41±0.5** | 1.91±0.2 | 10.33±0.5*** |
| #9 | 12 | 24.08±0.5* | 8.25±0.4** | 2.08±0.2 | 10.33±0.4*** |
| **35S::*MaFT8*/wt** | | | | | |
| #1 | 12 | 22.25±0.5*** | 7.83±0.4*** | 1.67±0.2 | 9.50±0.5*** |
| #2 | 12 | 22.67±0.6*** | 7.91±0.3*** | 1.91±0.2 | 9.83±0.4*** |
| #3 | 12 | 23.25±0.7** | 8.08±0.5*** | 1.83±0.2 | 9.91±0.5*** |
| #5 | 12 | 22.08±0.7*** | 7.91±0.5*** | 1.91±0.2 | 9.83±0.6*** |
| #6 | 12 | 23.17±0.8*** | 8.16±0.7*** | 1.83±0.2 | 10.00±0.6*** |
| #7 | 12 | 22.58±0.6*** | 8.08±0.5*** | 1.58±0.2 | 9.66±0.4*** |
| #8 | 12 | 23.08±0.9*** | 8.16±0.5*** | 1.83±0.2 | 9.75±0.6*** |
| #9 | 12 | 22.33±0.6*** | 8.25±0.4*** | 1.66±0.2 | 9.91±0.5*** |
| **35S::*MaFT9*/wt** | | | | | |
| #1 | 12 | 27.92±0.6 | 11.25±0.5 | 2.16±0.2 | 13.42±0.6 |
| #2 | 12 | 27.08±0.5 | 10.75±0.5 | 2.58±0.2 | 13.33±0.4 |
| #3 | 12 | 28.42±0.6 | 10.83±0.5 | 2.66±0.1 | 13.50±0.6 |
| #4 | 12 | 28.67±0.7 | 11.25±0.6 | 2.58±0.2 | 13.83±0.6 |
| #5 | 12 | 27.83±0.7 | 11.50±0.5 | 2.25±0.1 | 13.75±0.5 |
| #6 | 12 | 28.83±0.6 | 11.50±0.6 | 2.41±0.2 | 13.92±0.7 |
| #7 | 12 | 28.58±0.8 | 11.42±0.5 | 2.33±0.1 | 13.75±0.5 |
| #9 | 12 | 28.25±0.6 | 11.75±0.6 | 2.25±0.2 | 14.00±0.6 |
| **35S::*MaTSF1*/wt** | | | | | |
| #1 | 12 | 19.58±0.8*** | 5.84±0.5*** | 1.67±0.2 | 7.50±0.4*** |
| #2 | 12 | 20.92±0.8*** | 6.08±0.4*** | 1.75±0.2 | 7.84±0.5*** |
| #4 | 12 | 21.58±0.9*** | 6.17±0.4*** | 1.75±0.2 | 7.92±0.5*** |
| #6 | 12 | 19.84±0.7*** | 5.58±0.5*** | 1.84±0.2 | 7.42±0.5*** |
| #7 | 12 | 19.67±0.5*** | 5.84±0.4*** | 1.75±0.2 | 7.58±0.4*** |
| #9 | 12 | 21.34±0.9*** | 6.00±0.5*** | 1.67±0.2 | 7.67±0.6*** |
| **35S::*MaTSF2*/wt** | | | | | |
| #1 | 12 | 27.75±0.8 | 11.42±0.6 | 2.17±0.3 | 13.58±0.5 |
| #3 | 12 | 27.42±0.8 | 11.58±0.5 | 2.25±0.3 | 13.84±0.5 |
| #4 | 12 | 28.50±0.9 | 11.50±0.5 | 2.58±0.2 | 14.08±0.6 |
| #8 | 12 | 27.50±0.8 | 11.42±0.6 | 2.34±0.2 | 13.75±0.4 |
| #9 | 12 | 28.75±1.0 | 11.58±0.6 | 2.67±0.3 | 14.25±0.6 |
| #15 | 12 | 28.84±0.8 | 12.17±0.5 | 2.58±0.3 | 14.75±0.5 |
| #19 | 12 | 28.92±0.8 | 12.75±0.5 | 2.25±0.2 | 15.00±0.6 |
| #25 | 12 | 27.34±0.9 | 12.00±0.5 | 2.42±0.4 | 14.42±0.6 |

**Supplementary Table_S6**. List of oligonucleotide primers used in this study. (GW = genome walking).

| Name | Sequence | Purpose |
|---|---|---|
| FT-A9-F | GCACTGGCTGGTGacngayathcc | Gene isolation |
| FT-B6-R | GGACTCCCGCTGGcarttrwarwa | Gene isolation |
| FT-A19-F | GCGGACCTTCTACACCytngtnatggt | Gene isolation |
| MaFT1-F1 | ATTCAACACCAAGGACTTCTCCGCT | Gene completion |
| MaFT1- F2 | GGGGATCCCGTCGCTGCCATG | Gene completion |
| MaFT1-R1 | CTTGGTGTTGAAGTTCTGCCTCCAC | Gene completion |
| MaFT1-R2 | GCGTAGATCGTCTGCCGGACCGAT | Gene completion |
| MaFT2-F1 | ATTCAACACCAGGGACTTCGCCGAG | Gene completion |
| MaFT2-F2 | GGCTCGCCTGTGGCTGCCGTC | Gene completion |
| MaFT2-R1 | CCTGGTGTTGAAGTTTTGCCGCCAG | Gene completion |
| MaFT2-R2 | GTGTACACCGTCTGCCGACCCAAC | Gene completion |
| MaFT3-F1 | ATTCAGCACCAGAAGGTTCGTGCTG | Gene completion |
| MaFT3-F2 | ACCTGGCGCCTGTCGCCGCCAC | Gene completion |
| MaFT3-R1 | CTGGTGCTGAAGTTGTGGCGCATC | Gene completion |
| MaFT3-R2 | GCGAACACCGTCCCCCTCCCCATC | Gene completion |
| MaFT2-GW-F1 | GAAGAGACCAATCTAATCACTAGAAG | GW Intron specific |
| MaFT2-GW-F2 | ATGAGACGGATGCATGAAAGGAAGGA | GW Intron specific |
| MaFT2-GW-R1 | GCAAGTTAGCTTAAGCAGATATACTGG | GW Intron specific |
| MaFT2-GW-R2 | AATAGAGCAGCAGCGGCAGGGCATG | GW Intron specific |
| MaFT3-GW-F1 | ATAGGCATCTCTAGAATTTCTCGTCC | GW Intron specific |
| MaFT3-GW-F2 | GAAAAAGCTGCTGCTGATGATGATG | GW Intron specific |
| MaFT3-GW-R1 | CCAAGTTGCAGAAAACCTACGTACTC | GW Intron specific |
| MaFT3-GW-R2 | AAACCATCAGTTCCAGATCTATCAAC | GW Intron specific |
| MaFT4-GW-F1 | ACGCCAACATGACATCGTTCCTCAG | GW Intron specific |
| MaFT4-GW-F2 | ATTAACTGTGATGTTGGTGACACAGG | GW Intron specific |
| MaFT4-GW-R1 | CATCAGTTAAAGATCCGTCAGCTCT | GW Intron specific |
| MaFT4-GW-R2 | CGCTAAGGGACTCACCTAACGTGT | GW Intron specific |
| MaFT1-5’UTR-F | GTTGCCAGTGACTCCTGCAATCC | Full Gene amplification |
| MaFT1-3’UTR-R | TCAGCCATCCTCTTCACGGTCCACA | Full Gene amplification |
| MaFT1-0F | ATCTAGAATGTCGAGGGATCCTCTTG | Full Gene amplification |
| MaFT1-0R | ATCTAGACCACAGCCATTAGTACCTTCTTCC | Full Gene amplification |
| MaFT2-5’UTR-F | CTGACCATTTGCACCTCGAGAG | Full Gene amplification |
| MaFT2-3’UTR-R | GGGAAGGGAGGAGAAGTGTGA | Full Gene amplification |
| MaFT2-0F | ATCTAGAATGCAAAGAGATTCTTTGACTG | Full Gene amplification |
| MaFT2-0R | ATCTAGATGCAGGCCTCACCATAGC | Full Gene amplification |
| MaFT3-5’UTR-F | GAAGAAGCTTGTGCTCCTTCCTG | Full Gene amplification |
| MaFT3-3’UTR-R | GGCATGCATTTATTTACGGATTCA | Full Gene amplification |
| MaFT3-0F | ATCTAGAATGTCGAGGGACCCTCTG | Full Gene amplification |
| MaFT3-0R | ATCTAGAGTCGTCTCCCCGAAACCTT | Full Gene amplification |
| MaFT4-5’UTR-F | TGGAAAGAAGCTTCGGTCACAGA | Full Gene amplification |
| MaFT4-3’UTR-R | TGCCTTGAAACGCTAATACAGCTT | Full Gene amplification |
| MaFT4-0F | ATCTAGAATGTCGAGGGATCCGCTAAT | Full Gene amplification |
| MaFT4-0R | ATCTAGATATTGCTCTCAGCATCGGTC | Full Gene amplification |
| MaFT5-5’UTR-F | TCACTCACCATGAGTAGGGAGAC | Full Gene amplification |
| MaFT5-3’UTR-R | GGAGGAGGTGTACGAAATGAGAC | Full Gene amplification |
| MaFT5-0F | ATCTAGAATGAGTAGGGAGACTGATCCAC | Full Gene amplification |
| MaFT5-0R | ATCTAGAGTTCTGGAAGAGGTACATGCG | Full Gene amplification |
| MaFT6-5’UTR-F | TCAGTCCATTTGCACCCAGAGAA | Full Gene amplification |

| | | |
|---|---|---|
| MaFT6-3'UTR-R | CCTCACCAGAGCCTGTATTGTATA | Full Gene amplification |
| MaFT6-0F | ATCTAGAATGCAAAGAGACAGGGATTCTT | Full Gene amplification |
| MaFT6-0R | ATCTAGAGCATATCCAAGCTACATCCTC | Full Gene amplification |
| MaFT7-5'UTR-F | CCAGTAGCTCTTCTCGTCACTAAT | Full Gene amplification |
| MaFT7-3'UTR-R | TGAAAATTGTTATCTTAACTCTGTTGGTC | Full Gene amplification |
| MaFT7-0F | ATCTAGAATGTCGAGGGATCCGCTT | Full Gene amplification |
| MaFT7-0R | ATCTAGATCTGTTGGTCAAATCAAACGC | Full Gene amplification |
| MaFT8-5'UTR-F | GTCTCCTTTTTCTCTGTGTTGTTTGA | Full Gene amplification |
| MaFT8-3'UTR-R | CATTATGTTTTAGATGAAGTTGAGCTCT | Full Gene amplification |
| MaFT8-3'UTR-R1 | GCAGTCCAAAGAAGACCATCATCA | Full Gene amplification |
| MaFT8-0F | ATCTAGAATGTCGAGGGATCCGCTAGT | Full Gene amplification |
| MaFT8-0R | ATCTAGACTCTATTGATCTGATATGTAGTACC | Full Gene amplification |
| MaFT8-0R1 | ATCTAGAAGACCATCATCACATCCATCC | Full Gene amplification |
| MaFT9-5'UTR-F | TTACCGAGAAGCTCTCTTCTGCT | Full Gene amplification |
| MaFT9-3'UTR-R | AGATAATTTGCATGAGTTAGTTGAACCC | Full Gene amplification |
| MaFT9-3'UTR-R1 | CCCTAATGAGTATTGACTTACTCTC | Full Gene amplification |
| MaFT9-0F | ATCTAGAATGTCGAGGGACGCCCT | Full Gene amplification |
| MaFT9-0R | ATCTAGAGTTGAACCCTATTATTGTTCTGATG | Full Gene amplification |
| MaFT9-0R1 | ATCTAGACAGTCATCACGTCCATCCA | Full Gene amplification |
| MaFT10-5'UTR-F | ACCGTGGTGGTAGTTAGTCCATT | Full Gene amplification |
| MaFT10-3'UTR-R | CTTGGAAGTTTAGGCAGGTGTTATAT | Full Gene amplification |
| MaFT10-3'UTR-R1 | CCATAACCGTTGCAGTTTAAAGAAGA | Full Gene amplification |
| MaFT10-0F | ATCTAGAATGTCGAGGGACCCACTT | Full Gene amplification |
| MaFT10-0R | ATCTAGAATCTGATATACCTTCTTCCACCA | Full Gene amplification |
| MaFT10-0R1 | ATCTAGAAAGAAGACCATCACATCCATCCG | Full Gene amplification |
| MaFT11-5'UTR-F | CTGCGTACTAATGAGCAGGGAA | Full Gene amplification |
| MaFT11-3'UTR-R | AGTAGTGTTCAAGTGGCGAGTAG | Full Gene amplification |
| MaFT11-3'UTR-R1 | AGTAGTGTTCAAGTGGCGAGTAG | Full Gene amplification |
| MaFT11-0F | ATCTAGAATGAGCAGGGAAAGGGATC | Full Gene amplification |
| MaFT11-0R | ATCTAGACAGCTACATGGGATACATCC | Full Gene amplification |
| MaFT11-0R1 | ATCTAGACAGCTACATGGGATACATCC | Full Gene amplification |
| MaFT12-5'UTR-F | GAAGAAGTTTGTGCTCCTTCCTG | Full Gene amplification |
| MaFT12-3'UTR-R | GTATATCTCCTCTCAGCATCAGTCGTC | Full Gene amplification |
| MaFT12-0F | ATCTAGAATGTCGAGGGGCCCTCTG | Full Gene amplification |
| MaFT12-0R | ATCTAGAGTCGTCTCCCCGAAACCTT | Full Gene amplification |
| MaTSF1-5'UTR-F | *TTACAGCG*CAT*TAGCAGGCAG* | Full Gene amplification |
| Ma TSF1-3'UTR-R | CTAGATGATGGAGTTCTTAGTGTG | Full Gene amplification |
| Ma TSF1-0F | ATCTAGA*ATGTCACGAGGAAGGGATC* | Full Gene amplification |
| Ma TSF1-0R | ATCTAGAGTTCTTAGTGTGTTATTTACTACC | Full Gene amplification |
| MaTSF2-5'UTR-F | *CTTCTTCCATCACAGC*TTC*AACTTG*T | Full Gene amplification |
| Ma TSF2-3'UTR-R | TGATTCATGTCGTAAGCAATGACTTTTG | Full Gene amplification |
| Ma TSF2-0F | ATCTAGA*ATGCCCCAAGGAAGGGAT* | Full Gene amplification |
| Ma TSF2-0R | TGACTTTTGTTTATTACCTTCGTCCC | Full Gene amplification |
| MaFT1-qRT-F | CAATGGATCCGAGCTCAAAC | qRT-PCR |
| MaFT1-qRT-R | CACCAGCGTGTACAGAGTCC | qRT-PCR |
| MaFT2-qRT-F | GGCAGGAGATCGTGTGCTAT | qRT-PCR |
| MaFT2-qRT-R | GTTGTAGAGCTCGGCGAAGT | qRT-PCR |
| MaFT3-qRT-F | CCACAACTTCAGCACGAGAA | qRT-PCR |
| MaFT3-qRT-R | TCTCCTCTCAGCATCAGTCG | qRT-PCR |
| MaFT4-qRT-F | TTCGGTCACAGAATGTCGAG | qRT-PCR |
| MaFT4-qRT-R | CAGCAGACGGCTTGAACTC | qRT-PCR |
| MaFT5-qRT-F | CGCCAAAACTTCAACACCAG | qRT-PCR |

| | | |
|---|---|---|
| MaFT5-qRT-R | TGGAAGAGATACATGCGCCTA | qRT-PCR |
| MaFT6-qRT-F | ACGTCTTCGTGCTCTTCAGG | qRT-PCR |
| MaFT6-qRT-R | GCGATCCGAGGTTATAGAGC | qRT-PCR |
| MaFT7-qRT-F | GTTTGTGTTGTTCCGGCAAT | qRT-PCR |
| MaFT7-qRT-R | CCTTCTTCCACCACAACCAT | qRT-PCR |
| MaFT8-qRT-F | TCTCTGGAATCCATCGCTTT | qRT-PCR |
| MaFT8-qRT-R | GAACATGGCAGCAACAGGAT | qRT-PCR |
| MaFT9-qRT-F | GTGTTGTTCCGGCAGTCC | qRT-PCR |
| MaFT9-qRT-R | CCCCTAGGTTGTAAACTGCTG | qRT-PCR |
| MaFT11-qRT-F | CAACTCTGGGGATCCATCG | qRT-PCR |
| MaFT11-qRT-R | GTTGTAGAGCTCGGCGAAGT | qRT-PCR |
| MaFT12-qRT-F | TGGTTGCTTCGAGTGTCTTG | qRT-PCR |
| MaFT12-qRT-R | GCGGATGGTTTGAAGTCG | qRT-PCR |
| Ma TSF1-qRT-F | AGCCGGGATTCACCGTAT | qRT-PCR |
| Ma TSF1-qRT-R | CAGCAGGGAGTCCAAGGTTA | qRT-PCR |
| Ma TSF1-qRT-F | GCAGGGATTCATCGTATCGT | qRT-PCR |
| Ma TSF1-qRT-R | CCATTCTCTCTTTGGCAGTTG | qRT-PCR |
| RPS-qRT-F | CGGGATTGCCTGATATTGTG | qRT-PCR |
| RPS-qRT-R | CCCTCACAAATTGCGGATAC | qRT-PCR |
| AtAP1-qRT-F | AGGAGCAGTGGGATCAGCAG | qRT-PCR |
| AtAP1-qRT-F | TTGATACAGACCACCCATGTT | qRT-PCR |
| AtSAND-qRT-F | TTGATCCACTTGCAGACAAGGC | qRT-PCR |
| AtSAND-qRT-F | TACCCTTTGGCACACCTGATTG | qRT-PCR |

**Supplementary Table S7.** Accession numbers / Sequence identifiers used for phylogenetic analysis of *FT*/*TSF*-like gene family. (RGA, Rice Genome Annotation)

| **Plant** | **Common Name** | **Gene Name** | **Accession Numbers / Sequence Identifiers** | **Database** |
|---|---|---|---|---|
| *Arabidopsis thaliana* | Thale Cress | FT | NP_176726 | NCBI |
| *Arabidopsis thaliana* | Thale Cress | TSF | NP_193770 | NCBI |
| *Oryza sativa* | Rice | OsFTL1 | LOC_Os01g11940 | RGA |
| *Oryza sativa* | Rice | OsFTL2 (Hd3a) | LOC_Os06g06320 | RGA |
| *Oryza sativa* | Rice | OsFTL3 (RFT1) | LOC_Os06g06300 | RGA |
| *Oryza sativa* | Rice | OsFTL4 | LOC_Os09g33850 | RGA |
| *Oryza sativa* | Rice | OsFTL5 | LOC_Os02g39064 | RGA |
| *Oryza sativa* | Rice | OsFTL6 | LOC_Os04g41130 | RGA |
| *Oryza sativa* | Rice | OsFTL9 | LOC_Os01g54490 | RGA |
| *Oryza sativa* | Rice | OsFTL10 | LOC_Os05g44180 | RGA |
| *Oryza sativa* | Rice | OsFTL11 | LOC_Os11g18870 | RGA |
| *Oryza sativa* | Rice | OsFTL12 | LOC_Os06g35940 | RGA |
| *Oryza sativa* | Rice | OsFTL13 | LOC_Os02g13830 | RGA |
| *Zea mays* | Maize | ZCN7 | EU241899 | NCBI |
| *Zea mays* | Maize | ZCN8 | EU241900 | NCBI |
| *Zea mays* | Maize | ZCN12 | EU241903 | NCBI |
| *Zea mays* | Maize | ZCN13 | EU241904 | NCBI |
| *Zea mays* | Maize | ZCN14 | EU241905 | NCBI |
| *Zea mays* | Maize | ZCN15 | EU241906 | NCBI |
| *Zea mays* | Maize | ZCN16 | EU241907 | NCBI |
| *Zea mays* | Maize | ZCN17 | EU241908 | NCBI |
| *Zea mays* | Maize | ZCN18 | EU241909 | NCBI |
| *Zea mays* | Maize | ZCN19 | EU241910 | NCBI |
| *Zea mays* | Maize | ZCN20 | EU241911 | NCBI |
| *Zea mays* | Maize | ZCN21 | EU241912 | NCBI |
| *Zea mays* | Maize | ZCN24 | EU241914 | NCBI |
| *Zea mays* | Maize | ZCN25 | EU241915 | NCBI |
| *Zea mays* | Maize | ZCN25 | EU241916 | NCBI |
| *Allium cepa* | Onion | AcFT1 | KC485348 | NCBI |
| *Allium cepa* | Onion | AcFT2 | KC485349 | NCBI |
| *Allium cepa* | Onion | AcFT3 | KC485350 | NCBI |
| *Allium cepa* | Onion | AcFT4 | KC485351 | NCBI |
| *Allium cepa* | Onion | AcFT5 | KC485352 | NCBI |
| *Allium cepa* | Onion | AcFT6 | KC485353 | NCBI |
| *Hordeum vulgare* | Barley | HvFT1 | DQ100327 | NCBI |
| *Hordeum vulgare* | Barley | HvFT2 | DQ297407 | NCBI |
| *Hordeum vulgare* | Barley | HvFT3 | DQ411319 | NCBI |
| *Hordeum vulgare* | Barley | HvFT4 | DQ411320 | NCBI |
| *Hordeum vulgare* | Barley | HvFT5 | EF012202 | NCBI |

**Supplementary Table S8.** Accession numbers (genomic and mRNA) in the NCBI database for various banana *FT/TSF* genes identified in this study.

| Gene name | Accession No (genomic) | Accession No (mRNA) |
|---|---|---|
| ***MaFT1*** | KC296753 | KC296755 |
| ***MaFT2*** | JX842683 | JX842681 |
| ***MaFT3*** | JX842678 | HQ425707 |
| ***MaFT4*** | JX842677 | JX842676 |
| ***MaFT5*** | KF853462 | KF853467 |
| ***MaFT6*** | KF853463 | KF853468 |
| ***MaFT7*** | KF853464 | KF853469 |
| ***MaFT8*** | KF853465 | KF853470 |
| ***MaFT9*** | KF853466 | KF853471 |
| ***MaFT10*** | KM042900 | -- |
| ***MaFT11*** | KM042901 | KM054874 |
| ***MaFT12*** | KM042902 | KM054874 |
| ***MaTSF1*** | KM487739 | KF853460 |
| ***MaTSF2*** | KM487740 | KF853461 |

## Supplementary figures

**Figure S1. Phylogenetic analysis of Banana FT/TSF-like family proteins with Arabidopsis FT and TSF proteins.** The functional FT proteins of rice (Hd3a) and maize (ZCN8) are also included. The accession numbers are provided in methods.

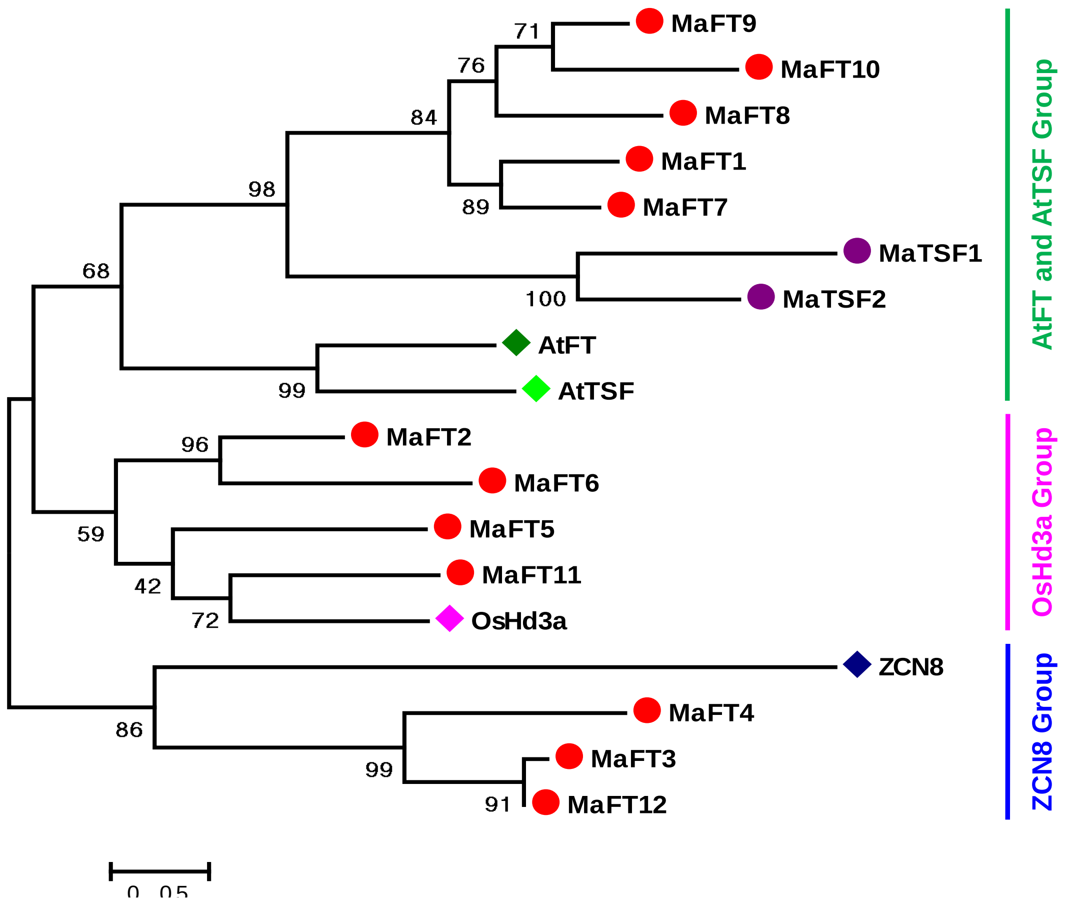


**Figure S1**

**Figure S2. Reversion of the late flowering phenotype of the Arabidopsis *ft-10* mutant upon ectopic expression of various banana *FT*/*TSF* genes.**

(A) Early flowering upon expression of *MaFT2*, *MaFT3*, *MaFT4*, *MaFT5* and *MaFT12* in *ft-10* mutant

(B) Early flowering upon expression of *MaFT1*, *MaFT8*, *MaTSF1* and *MaFT7* genes in *ft-10* mutant. The photographs A and B were taken 45 days and 38 days after planting respectively.

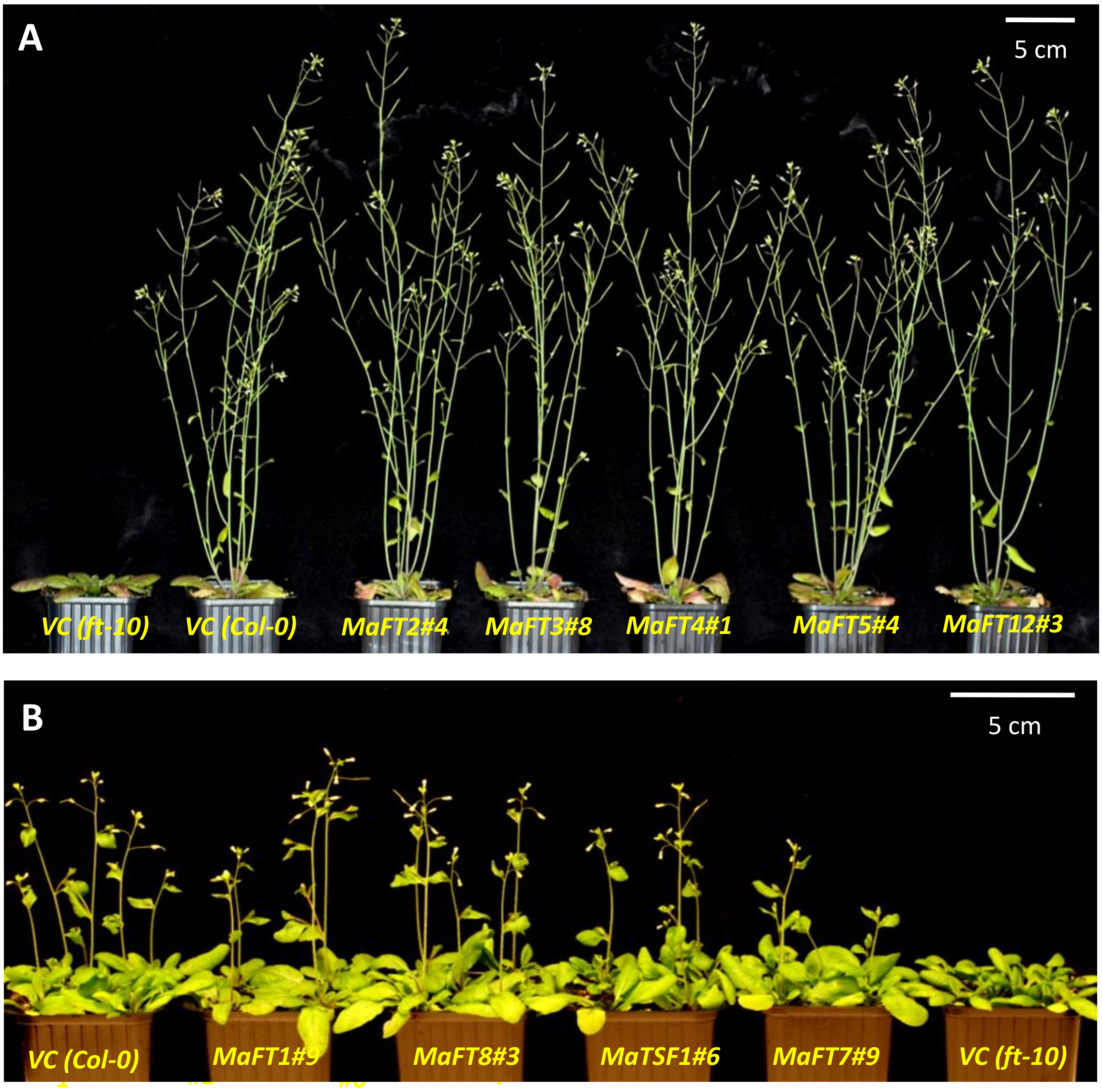


**Figure S2**

**Figure S3. Early flowering in Arabidopsis Col-0 induced by over-expression of various banana *FT*/*TSF*-like genes.** Except *MaFT6*, *MaFT9* and *MaTSF2*, all genes showed early flowering compared to vector control (VC) wild type plants. The photographs were taken 38 days after planting.

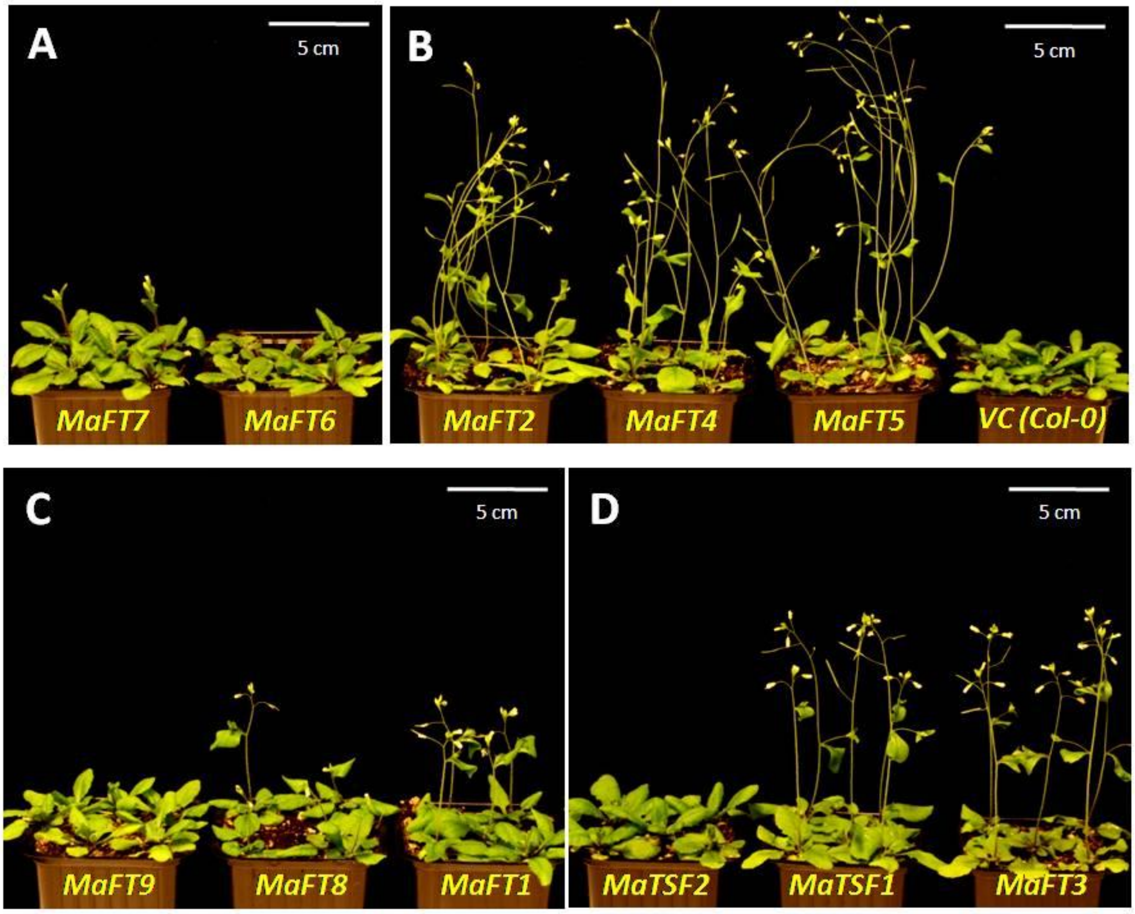


**Figure S3**

**Figure S4. Relative transcript level of *MaFT*/*TSF*-like genes in transgenic Arabidopsis lines ectopically expressing various banana genes.** The *SAND* (AT2G28390) gene of Arabidopsis is used as reference gene.

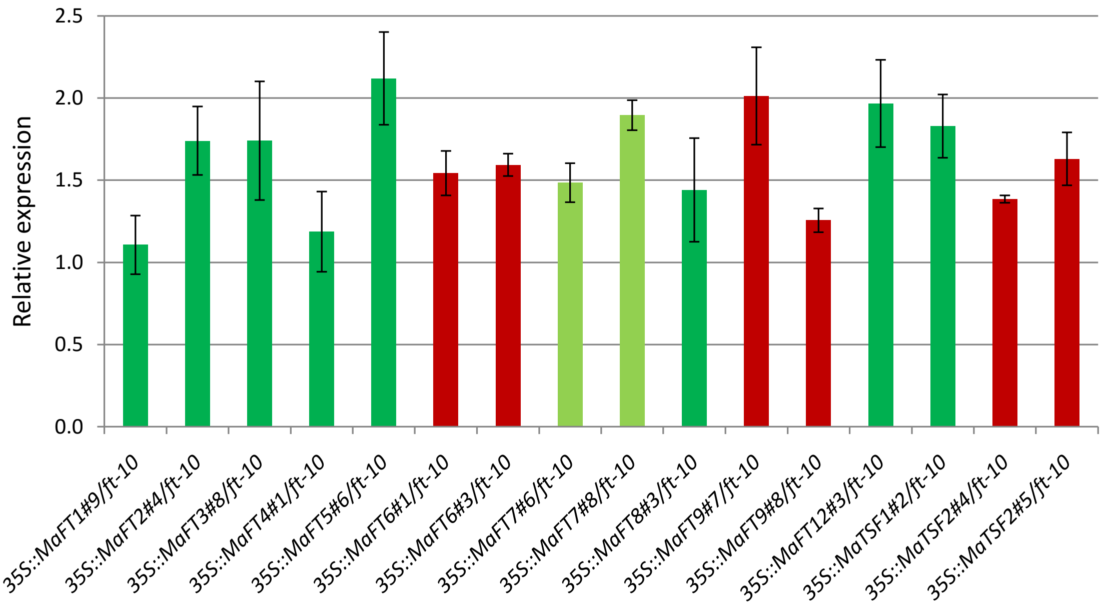


**Figure S4**

**Figure S5. Various field parameters during growth of banana**

Day length (top), Temperature (middle) and Humidity (bottom) (data taken from JISL weather station)

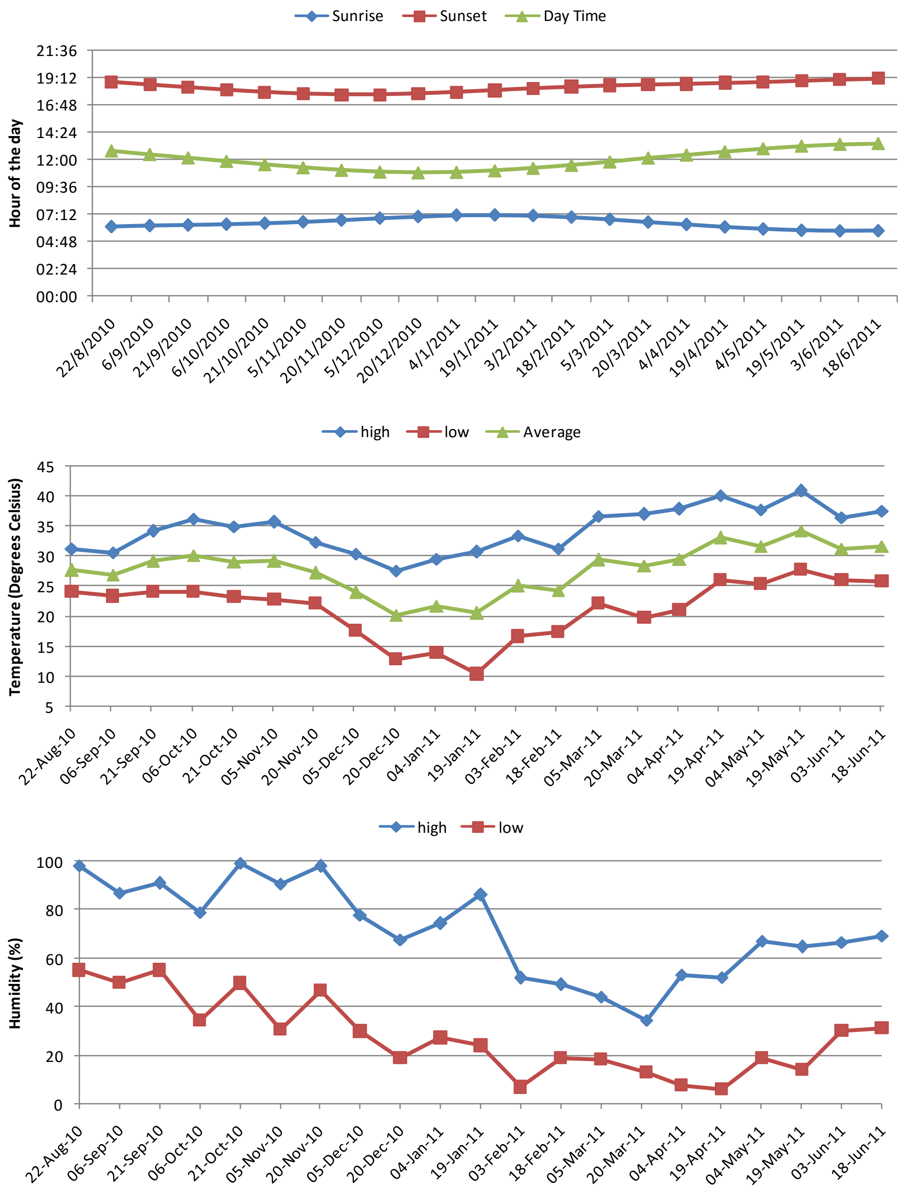


# Figure S5
